# Low MATR3 Expression Is Associated with Poor Prognosis in Endometrial Carcinoma: An Integrated Immunohistochemical and Transcriptomic Analysis

**DOI:** 10.3390/ijms27219708

**Published:** 2026-10-29

**Authors:** Jonasz Podemski, Dariusz Grzanka, Edyta Podemska, Natalia Skoczylas-Makowska, Dominika Jerka, Maciej Gagat, Paulina Antosik

**Affiliations:** 1 Department of Clinical Pathomorphology, Faculty of Medicine, Collegium Medicum in Bydgoszcz, Nicolaus Copernicus University in Torun, 85-094 Bydgoszcz, Poland; j.podemski@cm.umk.pl (J.P.); d_grzanka@cm.umk.pl (D.G.); edyta.trbk@gmail.com (E.P.); n.makowska@cm.umk.pl (N.S.-M.); 2 Department of Histology and Embryology, Collegium Medicum in Bydgoszcz, Nicolaus Copernicus University in Torun, 85-092 Bydgoszcz, Poland; dominika.jerka@cm.umk.pl (D.J.); mgagat@cm.umk.pl (M.G.)

**Keywords:** MATR3, matrin-3, endometrial carcinoma, prognostic biomarker, in silico analysis, co-expression, mitochondrial biogenesis

## Abstract

Endometrial carcinoma (EC) is the most common malignancy of the female genital tract, yet reliable prognostic biomarkers remain limited. Matrin-3 (MATR3), a nuclear matrix protein involved in genome stability and RNA processing, has been implicated in various cancers, but its role in EC has not been described. Here, we performed an integrated analysis combining immunohistochemical evaluation of MATR3 protein expression in a retrospective institutional cohort of 112 patients with EC and in silico analysis of *MATR3* mRNA expression in 172 TCGA cases. Immunohistochemical analysis revealed nuclear MATR3 expression in both EC and normal endometrial tissues, with significantly lower protein levels in EC compared to control tissues (*p* < 0.0001). *MATR3* mRNA was significantly reduced in EC tumour tissues compared to adjacent normal tissues (*p* < 0.0001). Low MATR3 protein expression was associated with shorter OS after adjustment for age, histological grade, pT, pN and pM in an LVSI-stratified Cox model (HR = 2.296; 95% CI: 1.284–4.104; *p* = 0.0050). Low *MATR3* mRNA expression was associated with improved overall survival in TCGA (median OS = 110.1 months; HR = 2.174; 95% CI: 1.066–4.43; *p* = 0.0303). Co-expression analysis identified *BCLAF1* and *TOP2B* as key positive correlates, while *TRAPPC5* and *FASTK* showed the strongest negative correlations. Genes positively correlated with *MATR3* mRNA expression were enriched for nuclear transcription and RNA-processing annotations, whereas negatively correlated genes were enriched for mitochondrial processes. These associations do not establish a mechanistic link between *MATR3* and pathway activity. Our findings support further investigation of MATR3 as a candidate prognostic marker in EC, with independent validation and functional studies required.

## 1. Introduction

Endometrial carcinoma (EC) is an important gynaecological malignancy. According to GLOBOCAN 2024 estimates, approximately 435,000 new cases of corpus uteri cancer occurred worldwide among women of all ages in 2024 [1]. These estimates refer to malignancies of the uterine corpus as a whole and are not restricted to endometrial carcinoma. A substantial burden of endometrial carcinoma is reported in highly developed countries [2,3]. Its incidence continues to rise, and projections from the International Agency for Research on Cancer indicate that the annual global burden could reach 608,130 new cases and 157,813 deaths by 2040 [4].

The five-year relative survival rate for endometrial carcinoma is highly unsatisfactory at 69% in regional metastatic EC and only 17% in EC with distant metastases, which is the result of too late a diagnosis [5,6,7]. So far, no effective EC early detection programme has been established [2,8]. Established risk factors for EC include advanced age, obesity, physical inactivity, prolonged endogenous or unopposed exogenous oestrogen exposure, insulin resistance, polycystic ovary syndrome, tamoxifen use, and hereditary predisposition, particularly Lynch syndrome [2]. Molecularly, EC is characterised by alterations affecting cell proliferation, survival, DNA repair, and genomic stability, including frequent changes in PTEN/PI3K/AKT, RAS/MAPK, and WNT/β-catenin signalling in endometrioid tumours and TP53 mutations with extensive copy-number alterations in serous and some high-grade endometrioid carcinomas [8,9,10,11]. TCGA further classified EC into four molecular subgroups: POLE ultramutated, microsatellite instability (MSI) hypermutated, copy-number low, and copy-number high [10,11]. Understanding the molecular changes and metabolic pathways associated with endometrial carcinoma may contribute to improving the current histological classification system and diagnostic testing methods, and establishing an efficient screening programme. It is crucial to find an appropriate marker that will improve diagnosis or serve as a starting point for modern targeted therapy [8,10]. Among numerous potential prognostic markers, the incompletely researched matrin-3 (MATR3) protein stands out. Its complex role in the cell is the subject of intensive scientific research, especially in the context of understanding its involvement in carcinogenesis processes.

MATR3 is a protein from the RNA-binding proteins (RBPs) family, initially identified in rat liver cells, belonging to a nuclear structure called the scaffold or nuclear matrix [12,13,14]. It plays an important role in regulating chromosome and genome integrity by interacting with DNA and/or DNA/RNA binding proteins. Additionally, it participates in the post-transcriptional regulation of mRNA, in the process of retaining excessively edited RNA in the nucleus, and in the phenomenon of silencing mRNA expression under the influence of miRNA [13,15]. Experimental studies have further shown that *MATR3* binds selected mRNA species and contributes to their stability [16]. Transcriptome-wide analyses have also identified *MATR3* as a regulator of alternative splicing, predominantly acting as a splicing repressor [17], while more recent data indicate that loss of *MATR3* promotes cryptic exon inclusion in multiple transcripts [18]. MATR3 influences the expression of genes associated with cell cycle arrest and modulation of apoptosis in response to cellular signals. It is involved in processes controlling cell survival, which may be important in the context of cancer development [12]. One of the key areas of interest is the role of MATR3 in the context of neurodegenerative diseases. Mutations in the *MATR3* gene have been identified in the context of diseases such as amyotrophic lateral sclerosis (ALS), familial frontotemporal dementia (FTD), and autosomal dominant distal myopathy with vocal cord and pharyngeal weakness [12,13,19,20]. Additionally, in basal-like breast cancer (BLBC), *MATR3* has been shown to act as a potential tumour suppressor gene [21]. Increased levels of MATR3 and phosphorylated MATR3 (P-MATR3) were detected in melanoma cells [22]. The role of this protein and its prognostic significance are still not fully understood in many cancers.

To our knowledge, the relationship between MATR3 and EC has not been described so far; therefore, the aim of this study was to evaluate the expression and prognostic significance of this protein in endometrial carcinoma. For this purpose, a collection of EC biopsies from our institution was subjected to immunohistochemical evaluation, and the results were correlated with clinical and pathological data as well as patient survival. Additionally, an in silico transcriptomic analysis was performed using TCGA data to characterise the molecular networks associated with *MATR3* mRNA expression in EC. These analyses were designed to identify expression patterns and functional annotations associated with MATR3 and to generate hypotheses for subsequent experimental investigation. They do not directly assess the effects of MATR3 on proliferation or invasion.

## 2. Results

### 2.1. MATR3 Protein and mRNA Expression in Endometrial Carcinoma and Normal Endometrial Tissues

**Figure 1:**
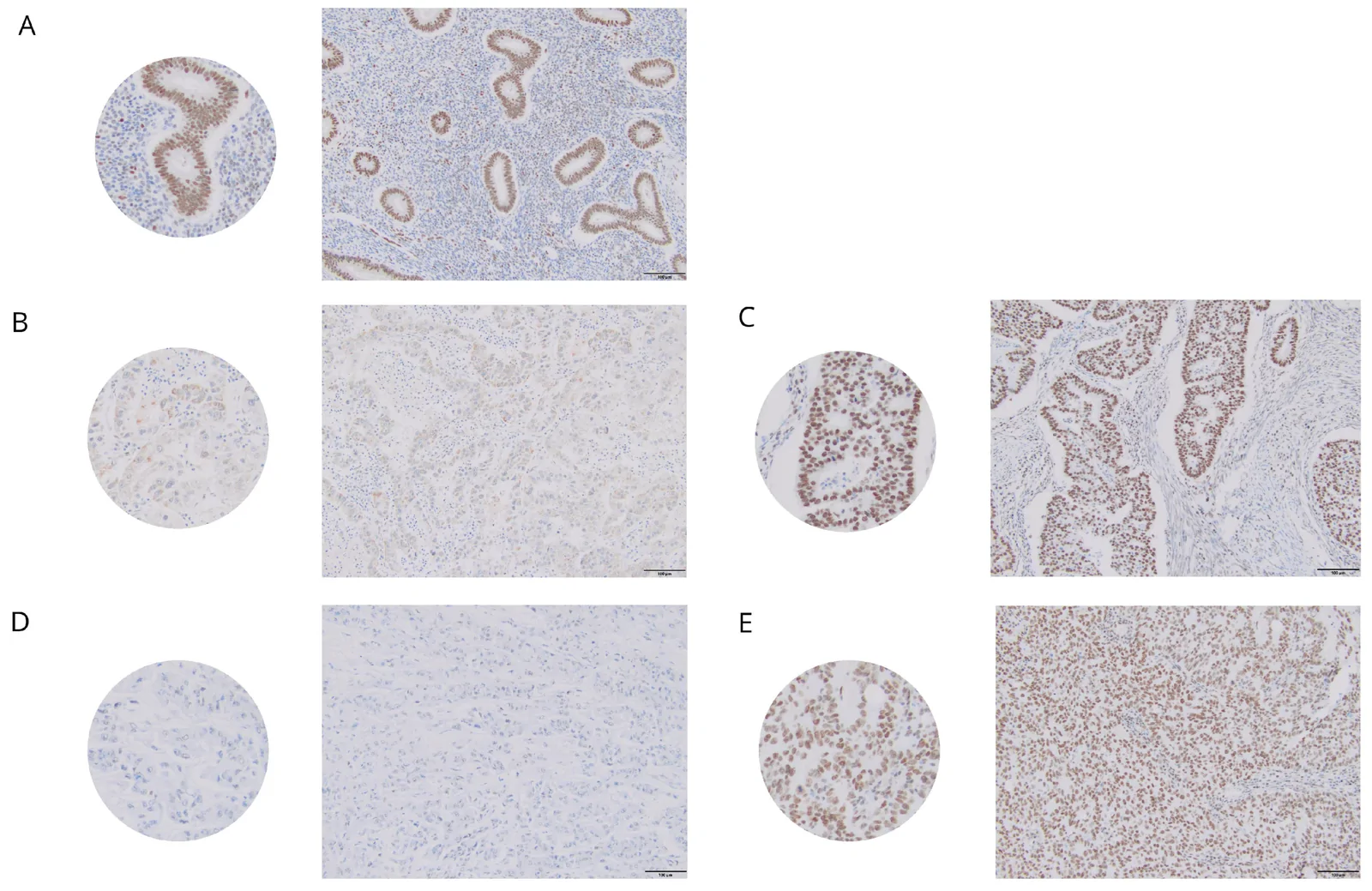
Representative immunohistochemical staining of MATR3 in endometrial tissues. (**A**) MATR3 expression in normal endometrium. (**B**) Low and (**C**) high MATR3 expression in endometrioid endometrial carcinoma. (**D**) Low and (**E**) high MATR3 expression in serous endometrial carcinoma.

Immunohistochemical staining revealed nuclear MATR3 expression in both endometrial carcinoma cells and normal endometrial cells. Representative microphotographs are shown in Figure 1. Agreement between the two independent H-score assessments was high across the 112 carcinoma cases, with an ICC(A,1) of 0.978 (95% CI 0.970–0.982). The reliability of the mean of the two assessments was 0.989 (ICC(A,2); 95% CI 0.985–0.991).

**Figure 2:**
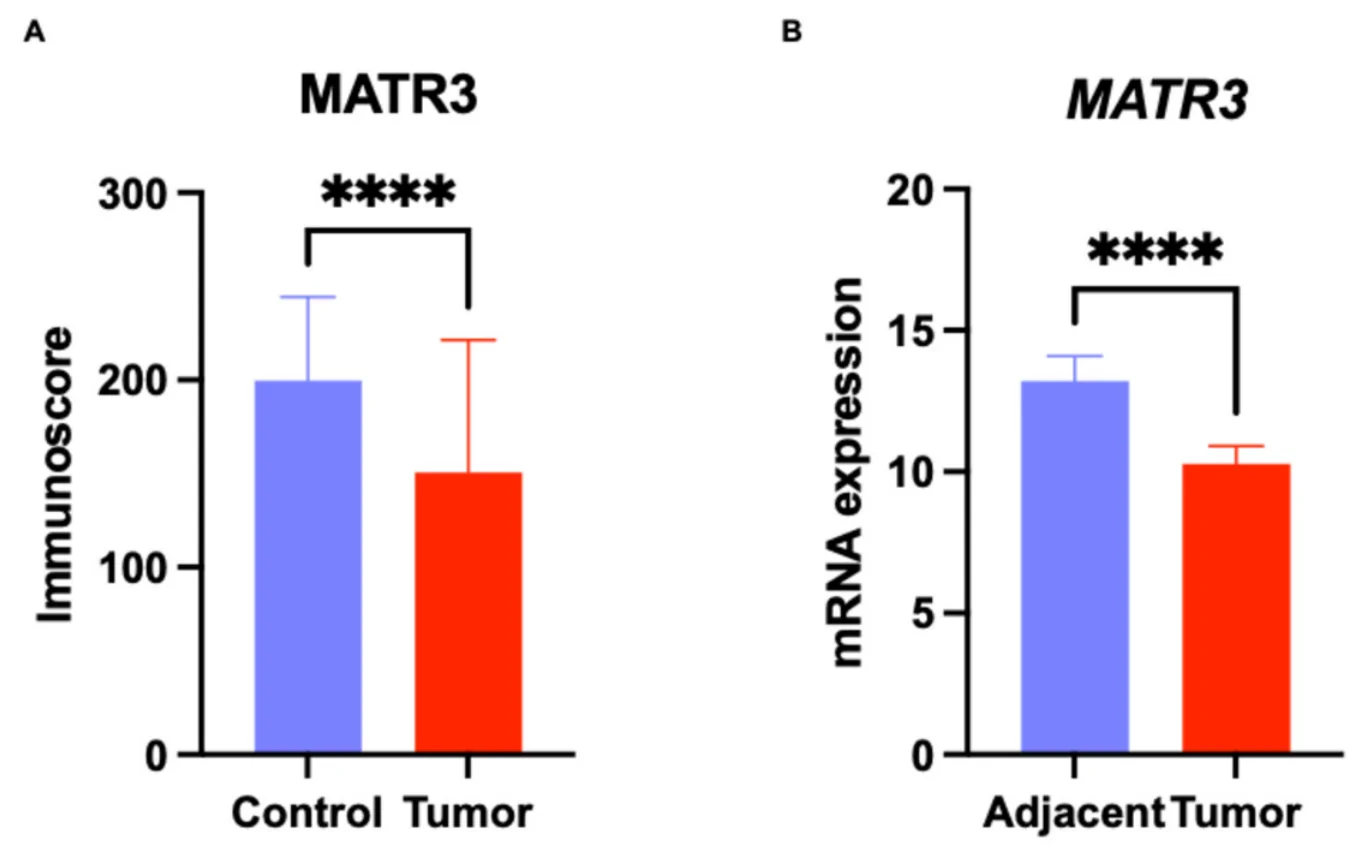
MATR3 expression in endometrial carcinoma. (**A**) MATR3 expression in the study cohort: control tissues, *n* = 31; endometrial carcinoma, *n* = 112. (**B**) *MATR3* mRNA expression in the in silico cohort: adjacent tissues, *n* = 135; endometrial carcinoma, *n* = 172. Asterisks indicate statistical significance: **** *p* < 0.0001.

MATR3 protein expression was assessed in control endometrial samples and endometrial carcinoma tissues. The median MATR3 H-score was 190.00 in the control group (*n* = 31) and 146.00 in the endometrial carcinoma group (*n* = 112). MATR3 protein expression was significantly lower in carcinoma tissues than in control tissues (two-sided Mann–Whitney U test, *p* < 0.0001; Figure 2A).

*MATR3* mRNA expression was also analysed in an independent in silico dataset. The median *MATR3* mRNA level was 12.72 in adjacent non-tumour tissues (*n* = 135) and 10.28 in endometrial carcinoma tissues (*n* = 172). *MATR3* mRNA expression was significantly lower in cancer samples than in adjacent tissues (*p* < 0.0001; Figure 2B). The comparative analysis of *MATR3* mRNA expression between histological subtypes of endometrial carcinoma in both the immunohistochemical cohort and the in silico cohort is presented in Supplementary Figure S1.

### 2.2. Associations of MATR3 Protein and mRNA Expression with Clinicopathological Parameters

The associations between MATR3 expression and clinicopathological parameters are presented in Table 1. Low MATR3 expression was observed in 62 cases (55.35%), whereas high MATR3 expression was found in 50 cases (44.65%).

In the institutional cohort, MATR3 expression was associated with pT status and pN status at the nominal significance level. Low MATR3 expression was more frequent in tumours classified as pT3–T4 than in pT1–T2 cases (nominal *p* = 0.0252) and in pN1 than in pN0 cases (80.00% vs. 50.00%; nominal *p* = 0.0238). An association with FIGO stage also reached nominal significance (*p* = 0.0295; Table 1). However, none of these associations remained statistically significant after Benjamini–Hochberg adjustment across the nine clinicopathological comparisons (adjusted *p* = 0.0885 for each; Supplementary Table S1). No significant associations were found between MATR3 expression and age, histological grade, pM status, lymphovascular invasion, histological type, or myometrial invasion.

Among the 172 patients in the TCGA in silico cohort, 97 (56.40%) showed high *MATR3* mRNA expression and 75 (43.60%) showed low expression. The relationship between *MATR3* mRNA expression and clinicopathological features is summarized in Table 2. High expression was observed in 60.38% of patients aged ≤60 years and 54.62% of those aged >60 years, with no significant association with age (nominal *p* = 0.4228). Analysis by histological grade revealed high expression in 53.85% of G1, 52.38% of G2, and 57.25% of G3 cases (nominal *p* = 0.8991). With respect to histological type, high expression was identified in 57.84% of endometrioid and 54.29% of non-endometrioid carcinomas (nominal *p* = 0.6439). None of these associations was statistically significant before or after Benjamini–Hochberg adjustment across the three clinicopathological comparisons (adjusted *p* = 0.8991 for each; Supplementary Table S1B).

### 2.3. Prognostic Associations of MATR3 Protein and mRNA Expression

**Figure 3:**
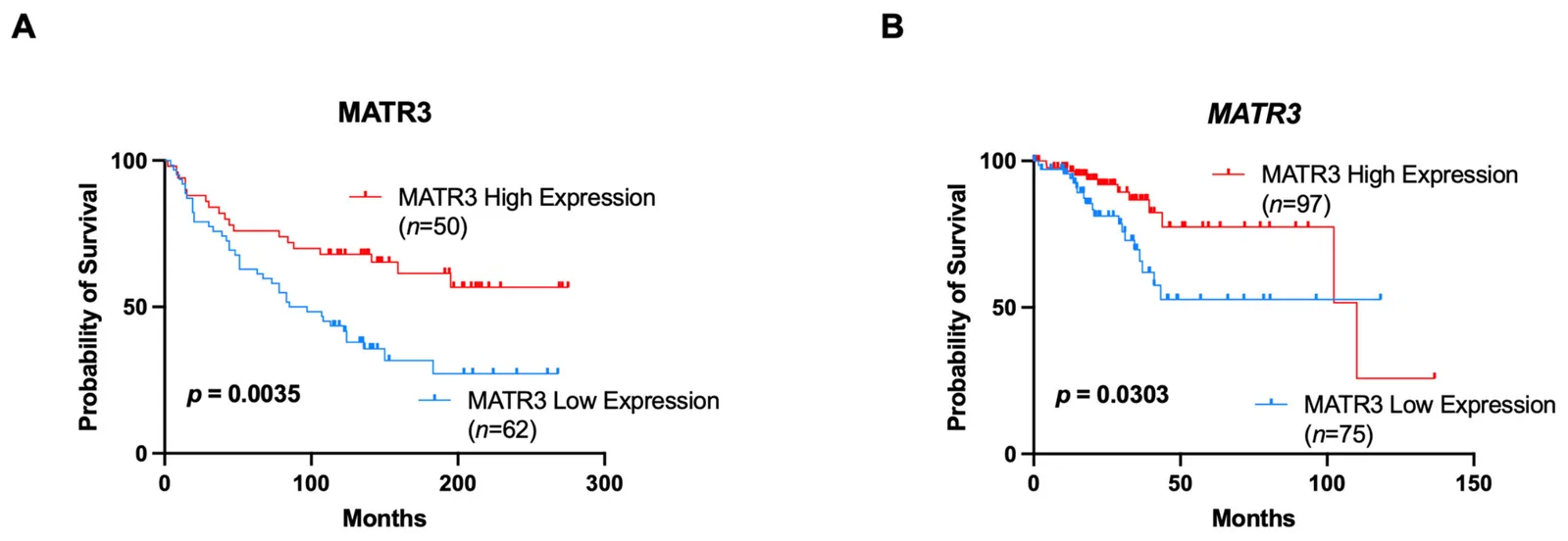
Kaplan–Meier curves illustrating OS in EC patients stratified by (**A**) MATR3 protein expression and (**B**) *MATR3* mRNA expression. Abbreviations: OS, overall survival; HR, hazard ratio; CI, confidence interval; EC, endometrial carcinoma; MATR3, matrin-3; mRNA, messenger RNA.

In the institutional IHC cohort, the Kaplan–Meier estimates indicated poorer overall survival (OS) among patients with low MATR3 protein expression (Figure 3A). The median OS in this group was 91.0 months, while the median was not reached among patients with high expression. The survival difference remained statistically significant after Benjamini–Hochberg adjustment across the two overall-cohort log-rank comparisons (nominal *p* = 0.0035; adjusted *p* = 0.0070). The log-rank-derived hazard ratio for low versus high expression was 2.181 (95% CI: 1.315–3.618).

Kaplan–Meier survival analysis demonstrated that high *MATR3* mRNA expression was significantly associated with improved overall survival (OS) in EC patients compared to the low-expression group (Figure 3B). In the TCGA cohort, the median OS was 110.1 months in the high-*MATR3*-mRNA-expression group, whereas the median OS was not reached in the low-expression group during the available follow-up. This difference was statistically significant by the log-rank test (nominal *p* = 0.0303; Benjamini–Hochberg-adjusted *p* = 0.0303). The hazard ratio (HR) calculated using the log-rank method indicated that patients with low *MATR3* expression had over twice the risk of death compared to those with high expression (HR = 2.174; 95% CI: 1.066–4.43).

**Figure 4:**
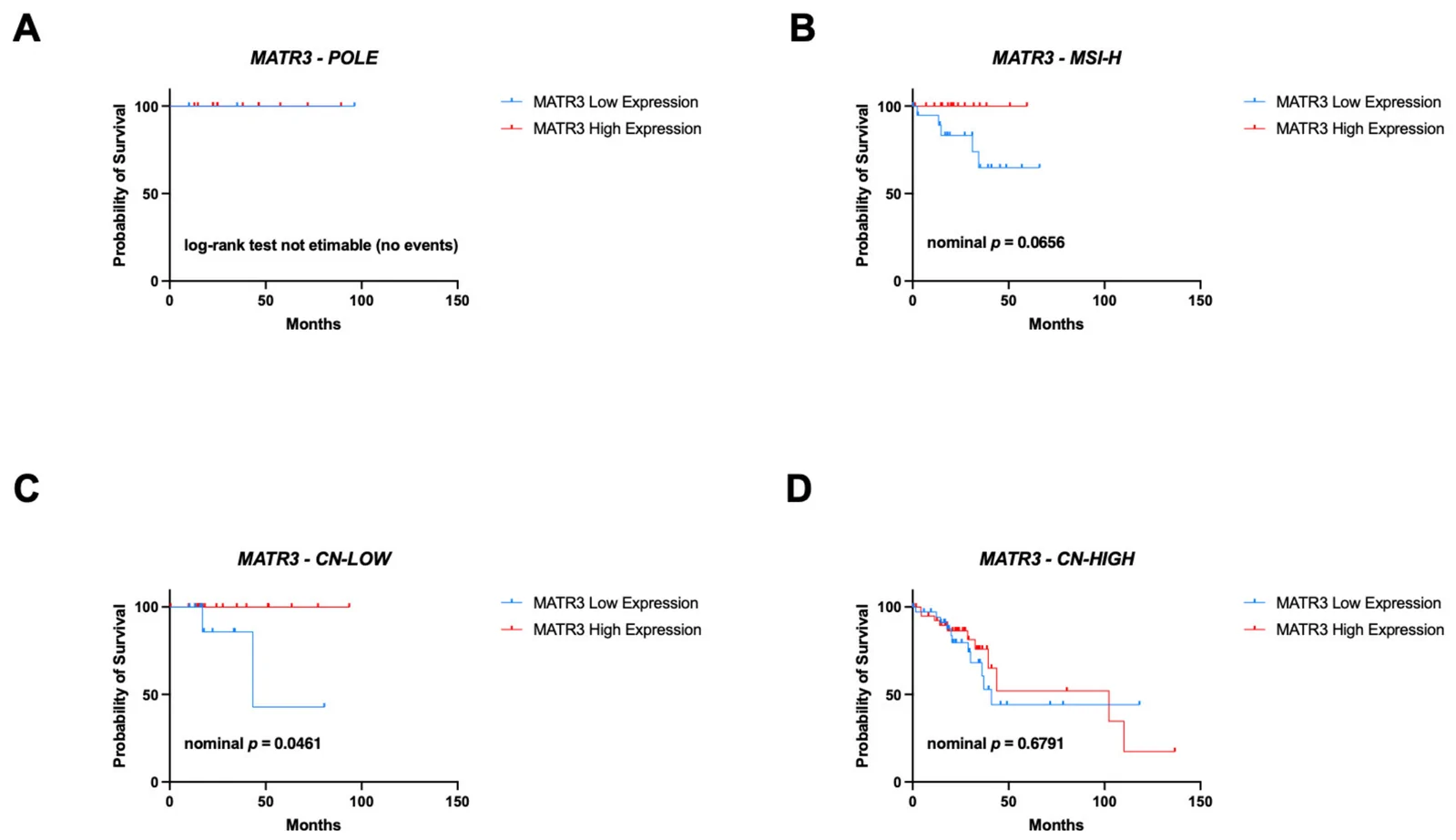
Kaplan–Meier curves for overall survival according to *MATR3* mRNA expression in TCGA-UCEC molecular subtypes: (**A**) POLE, (**B**) MSI-H, (**C**) CN-low and (**D**) CN-high. Nominal log-rank *p*-values and Benjamini–Hochberg-adjusted *p*-values for the three estimable comparisons are reported in Supplementary Table S2. No deaths occurred in the POLE subgroup; therefore, its log-rank *p*-value was not estimable. None of the estimable comparisons met an adjusted *p*-value threshold of 0.05. Abbreviations: OS, overall survival; TCGA-UCEC, The Cancer Genome Atlas uterine corpus endometrial carcinoma cohort; POLE, DNA polymerase epsilon; MSI-H, microsatellite instability-high; CN-low, copy-number low; CN-high, copy-number high; MATR3, matrin-3; mRNA, messenger RNA.

Subtype-stratified Kaplan–Meier survival analysis was performed to evaluate the association between *MATR3* mRNA expression and OS within the TCGA molecular subgroups of EC. Molecular subtype annotation was available for 166 of 172 TCGA-UCEC cases, including CN-high tumours (*n* = 82), MSI-H tumours (*n* = 39), CN-low tumours (*n* = 30), and POLE-mutated tumours *(n* = 15). In the CN-low subgroup, high *MATR3* mRNA expression was associated with improved OS at the nominal significance level (log-rank *p* = 0.0461; Figure 4C), but this association did not remain significant after Benjamini–Hochberg adjustment across the three estimable subtype comparisons (adjusted *p* = 0.0983). In this subgroup, the median OS was 43.3 months in patients with low *MATR3* expression, whereas it was not reached in the high-expression group. No statistically significant survival differences were observed in the MSI-H subgroup (nominal *p* = 0.0656; adjusted *p* = 0.0983; Figure 4B) or the CN-high subgroup (nominal and adjusted *p* = 0.6791; Figure 4D). No deaths occurred in the POLE subgroup, precluding estimation of a log-rank *p*-value (Figure 4A). Overall, none of the estimable subtype comparisons remained statistically significant after adjustment. Patient numbers, event counts, and nominal and adjusted *p*-values are presented in Supplementary Table S2. All 166 patients with available molecular subtype annotations contributed to the subtype-specific survival analyses, including all 30 CN-low and 15 POLE-mutated cases. Only two deaths occurred in the CN-low subgroup, both in the low-expression group. A finite hazard ratio could not be estimated using standard Cox regression for CN-low or MSI-H tumours because no deaths occurred in their high-expression groups; no hazard ratio could be estimated for POLE tumours because there were no deaths in either group. For CN-high tumours, the univariable hazard ratio for low versus high *MATR3* expression was 1.19 (95% CI, 0.51–2.77). These subtype-specific analyses are exploratory and do not establish subtype-specific prognostic value.

*MATR3* mRNA expression was also compared across the four TCGA molecular subtypes using the Kruskal–Wallis test. No statistically significant differences were observed among POLE, MSI-H, CN-low, and CN-high tumours (H = 4.5811, *p* = 0.2052). The results of this expression analysis are presented in Supplementary Table S3 and Supplementary Figure S2.

DNA methylation analysis of *MATR3* included 34 MATR3-associated CpG probes in the TCGA-UCEC cohort. After merging methylation beta values with *MATR3* mRNA expression data, 168 cases with complete data were included in the correlation analysis.

Pearson correlation analysis identified two CpG probes with statistically significant negative correlations with *MATR3* mRNA expression: cg14717906 (r = −0.172, *p* = 0.026) and cg04447756 (r = −0.158, *p* = 0.041). According to the GRCh38 genomic coordinates and transcript annotations available in MEXPRESS, cg14717906 (chr5:139306176) and cg04447756 (chr5:139309297) are located within introns 1 and 2, respectively, of MATR3 transcript ENST00000394805. These sites therefore have an intragenic location relative to this transcript. Their potential overlap with regulatory regions associated with alternative transcripts was not functionally assessed.

Additional analysis using the MEXPRESS platform showed that the negative association between cg04447756 methylation and *MATR3* expression remained statistically significant after Benjamini–Hochberg correction (*p* = 0.00044, adjusted *p* = 0.023). In contrast, cg14717906 was not significant in the additional MEXPRESS analysis (*p* = 0.41). Detailed results of the methylation analysis are provided in Supplementary Figures S3–S5.

In univariable Cox regression analyses, low MATR3 expression was significantly associated with shorter overall survival (OS) in patients with endometrial carcinoma in our cohort (HR 2.223, 95% CI 1.284–3.848, *p* = 0.0043; Table 3). Significant associations with shorter OS were also observed for age > 60 years (HR 3.275, 95% CI 1.658–6.468, *p* = 0.0006), histological grade G3 versus G1–G2 (HR 2.166, 95% CI 1.304–3.598, *p* = 0.0028), and the presence of LVSI (HR 2.765, 95% CI 1.569–4.871, *p* = 0.0004). The associations of pT, pN, and pM status with OS did not reach statistical significance.

In the multivariable Cox model stratified by LVSI status, low MATR3 expression remained significantly associated with shorter OS after adjustment for age, histological grade, pT, pN, and pM (HR 2.296, 95% CI 1.284–4.104, *p* = 0.0050; Table 3). Age > 60 years was also significantly associated with shorter OS (HR 3.272, 95% CI 1.614–6.633, *p* = 0.0010), whereas histological grade, pT, pN, and pM did not reach statistical significance. LVSI was incorporated as a stratification variable to accommodate evidence of non-proportional hazards; consequently, no adjusted HR was estimated for LVSI.

Adding MATR3 expression to the LVSI-stratified clinical model improved model fit (likelihood-ratio χ^2^ = 8.243, df = 1, nominal *p* = 0.0041). AIC decreased from 434.029 for the clinical model to 427.786 for the model additionally incorporating MATR3. Apparent Harrell’s C, evaluated within LVSI strata, increased from 0.6917 to 0.7232 (ΔC = 0.0315; Supplementary Table S4).

### 2.4. Co-Expression Analysis: Top Correlated Genes

Spearman’s rank correlation analysis revealed that *BCLAF1* and *TOP2B* are the most strongly positively correlated genes with *MATR3* mRNA expression, exhibiting coefficients of ρ = 0.785 (*p* = 2.98 × 10^−111^) and ρ = 0.745 (*p* = 2.31 × 10^−94^), respectively (Table 4). In contrast, *TRAPPC5* and *FASTK* show the strongest negative correlations with *MATR3*, with ρ = −0.690 (*p* = 1.04 × 10^−75^) and ρ = −0.687 (*p* = 9.41 × 10^−75^), respectively (Table 5).

### 2.5. Functional Enrichment Analysis

**Figure 5:**
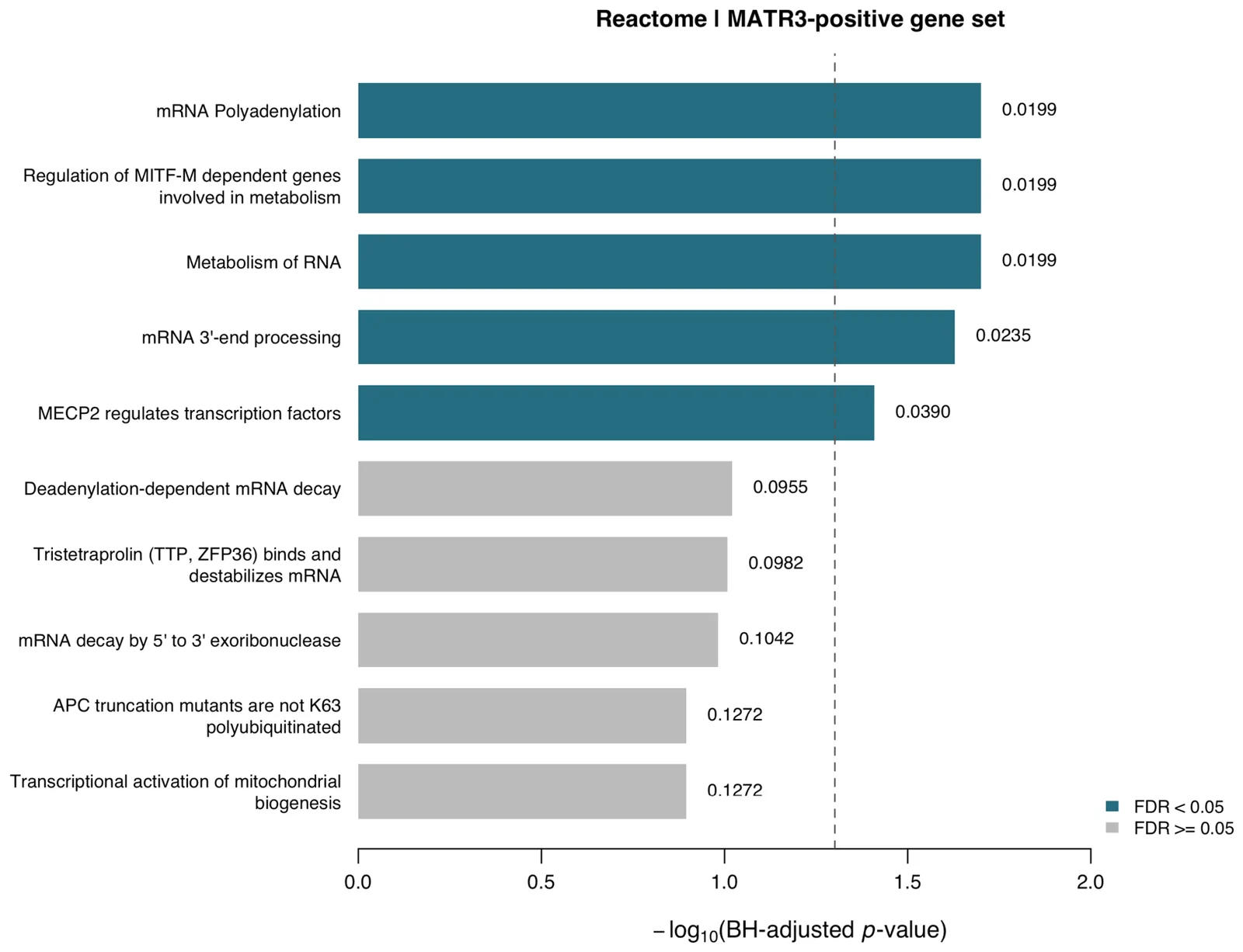
Reactome enrichment analysis of the 50 genes positively correlated with *MATR3*. The ten pathways with the lowest nominal *p*-values are displayed. Bar lengths represent −log10(Benjamini–Hochberg-adjusted *p*-values), with adjusted *p*-values shown beside the bars. Blue bars indicate adjusted *p* < 0.05; grey bars indicate adjusted *p* ≥ 0.05. The dashed line marks the adjusted *p* = 0.05 threshold. Reactome release 97 was accessed on 19 October 2026.

Reactome analysis identified five pathways enriched among genes positively correlated with *MATR3* mRNA expression after Benjamini–Hochberg adjustment. These included mRNA polyadenylation, regulation of MITF-M-dependent genes involved in metabolism and metabolism of RNA (adjusted *p* = 0.0199 for each), mRNA 3′-end processing (adjusted *p* = 0.0235), and MECP2-mediated regulation of transcription factors (adjusted *p* = 0.0390; Figure 5). Deadenylation-dependent mRNA decay reached nominal significance but did not meet the adjusted threshold (nominal *p* = 0.0017; adjusted *p* = 0.0955).

**Figure 6:**
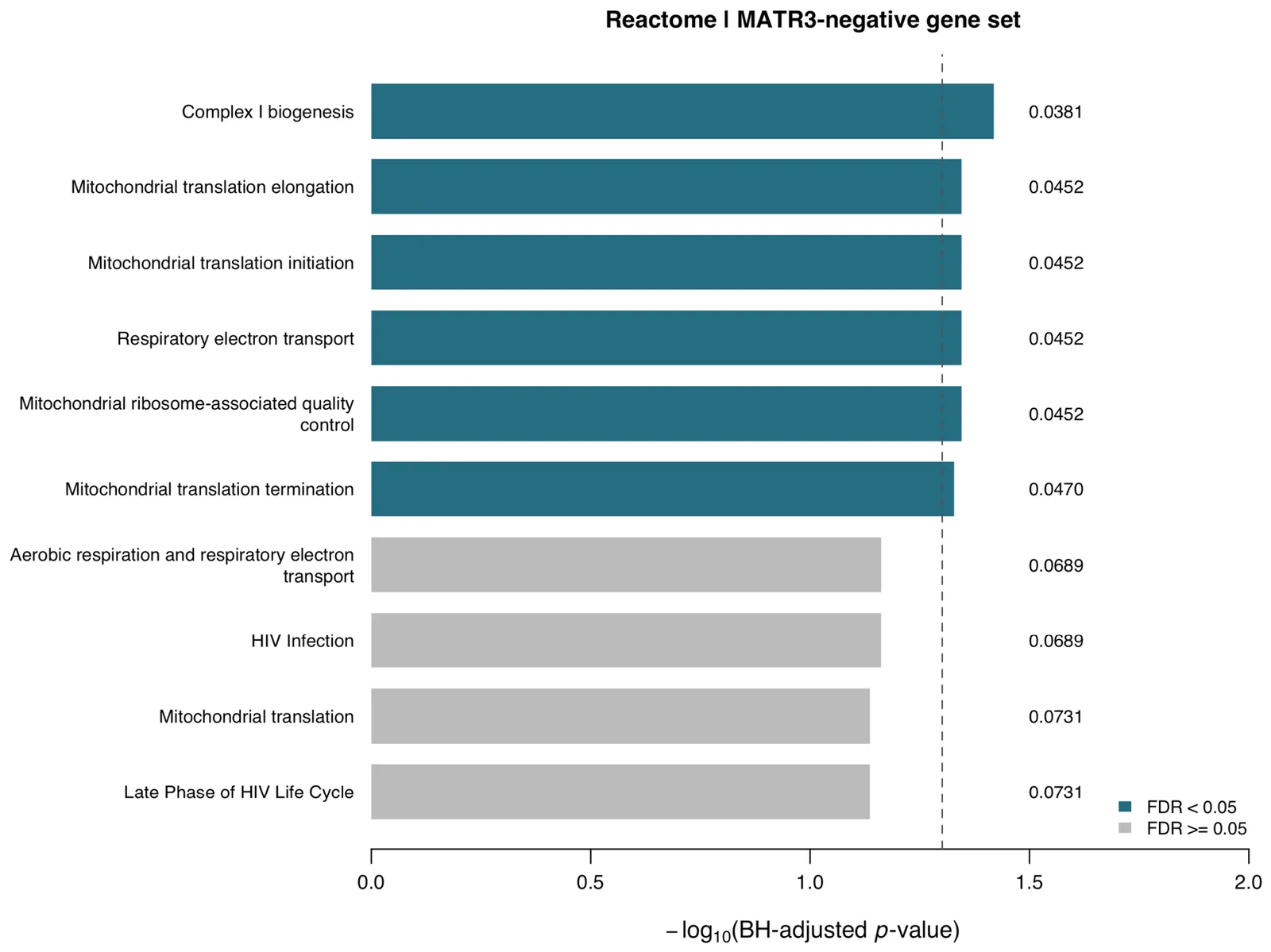
Reactome enrichment analysis of the 50 genes negatively correlated with *MATR3*. The ten pathways with the lowest nominal *p*-values are displayed. Bar lengths represent −log10(Benjamini–Hochberg-adjusted *p*-values), with adjusted *p*-values shown beside the bars. Blue bars indicate adjusted *p* < 0.05; grey bars indicate adjusted *p* ≥ 0.05. The dashed line marks the adjusted *p* = 0.05 threshold. Reactome release 97 was accessed on 19 October 2026.

Among genes negatively correlated with *MATR3* mRNA expression, six pathways remained significant after adjustment: complex I biogenesis (adjusted *p* = 0.0381), mitochondrial translation elongation, mitochondrial translation initiation, respiratory electron transport and mitochondrial ribosome-associated quality control (adjusted *p* = 0.0452 for each), and mitochondrial translation termination (adjusted *p* = 0.0470; Figure 6). These results indicate enrichment of RNA-related and mitochondrial pathway annotations in the respective gene sets. Detailed results are provided in Supplementary Table S5A.

### 2.6. Gene Ontology Enrichment Analysis

**Figure 7:**
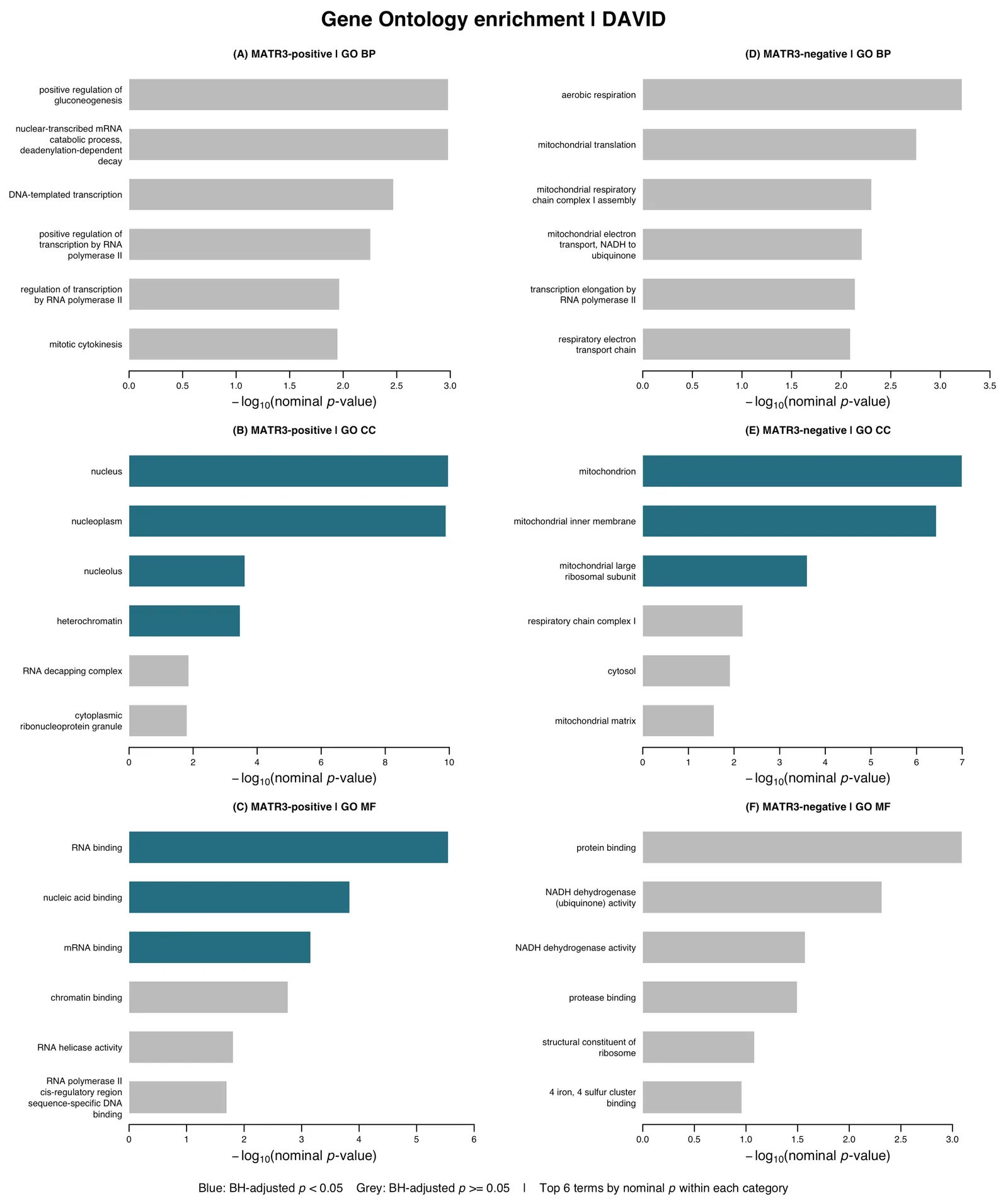
Gene Ontology enrichment analysis of genes correlated with *MATR3* mRNA expression using DAVID (Knowledgebase v2026_2; accessed 19 October 2026). Panels (**A**–**C**) show biological process (BP), cellular component (CC) and molecular function (MF) results for positively correlated genes; panels (**D**–**F**) show the corresponding categories for negatively correlated genes. Each panel displays the six terms with the lowest nominal *p*-values. Bar lengths represent −log10(nominal *p*-values). Blue bars identify terms with Benjamini–Hochberg-adjusted *p* < 0.05, and grey bars identify terms that did not meet this threshold. Adjustment was performed within each GO category and gene set.

After Benjamini–Hochberg adjustment, the positively correlated gene set showed enrichment of four cellular component terms: nucleus and nucleoplasm (adjusted *p* = 8.05 × 10^−9^ for each), nucleolus (adjusted *p* = 0.0102) and heterochromatin (adjusted *p* = 0.0109; Figure 7B). Three molecular function terms remained significant: RNA binding (adjusted *p* = 4.73 × 10^−4^), nucleic acid binding (adjusted *p* = 0.0124) and mRNA binding (adjusted *p* = 0.0391; Figure 7C). No biological process term met the adjusted threshold in this gene set (Figure 7A).

In the negatively correlated gene set, significant enrichment was confined to the cellular component category: mitochondrion (adjusted *p* = 1.15 × 10^−5^), mitochondrial inner membrane (adjusted *p* = 2.10 × 10^−5^) and mitochondrial large ribosomal subunit (adjusted *p* = 0.0095; Figure 7E). No biological process or molecular function terms remained significant after adjustment (Figure 7D,F). In particular, aerobic respiration and protein binding reached nominal significance but not the adjusted threshold (adjusted *p* = 0.1721 and 0.0983, respectively). Results are summarised in Supplementary Table S5B,C.

### 2.7. Protein–Protein Interaction Network Analysis

**Figure 8:**
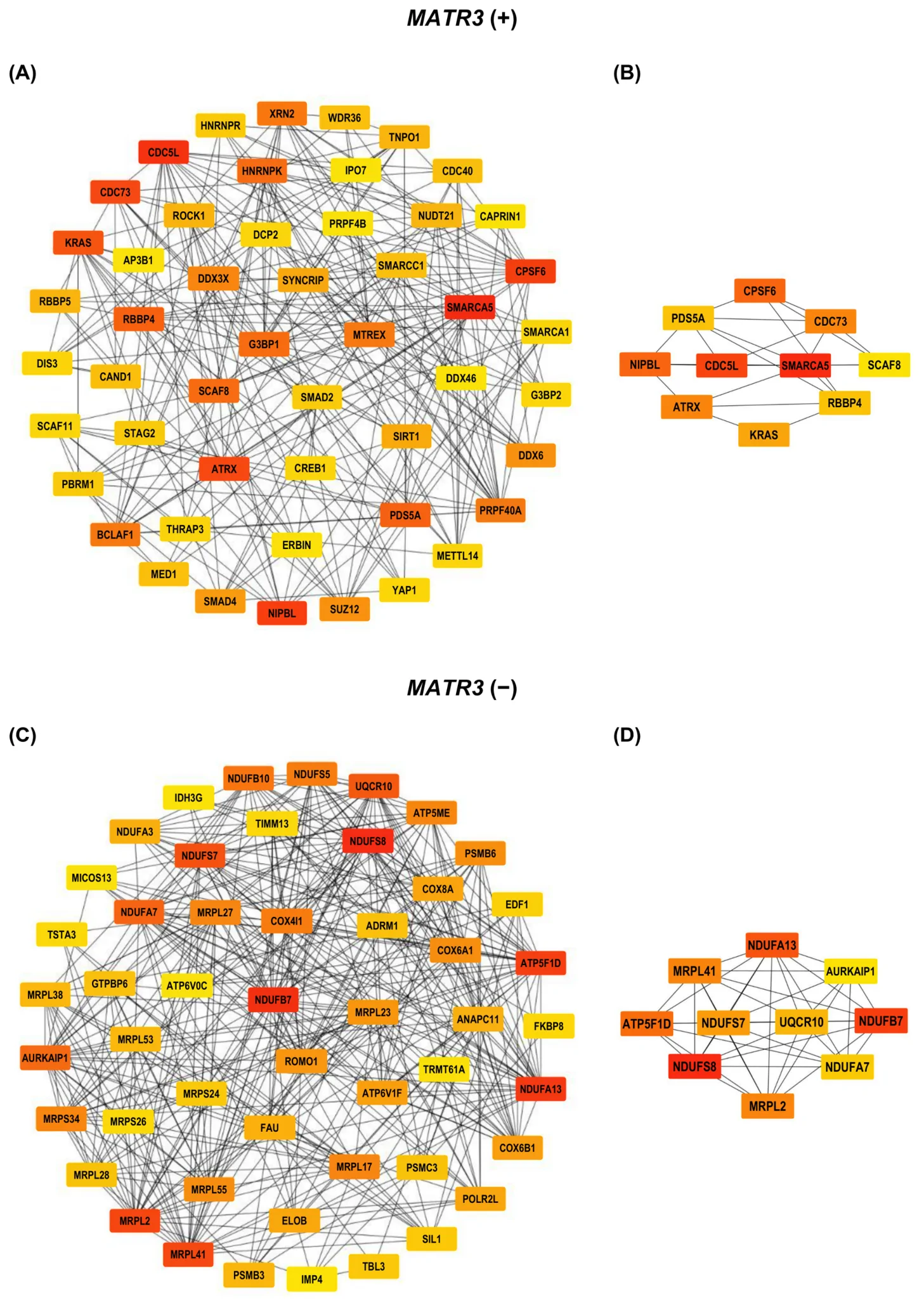
STRING-derived protein–protein interaction (PPI) networks for proteins encoded by the 50 genes most strongly positively correlated and the 50 genes most strongly negatively correlated with MATR3 mRNA expression in endometrial carcinoma, visualised in Cytoscape. (**A**,**C**) Networks containing the proteins encoded by all 50 genes in the positively (**A**) and negatively (**C**) correlated sets. (**B**,**D**) Subnetworks highlighting the ten hub proteins with the highest degree centrality within the corresponding networks. Edges represent STRING-derived protein associations. STRING integrates evidence from multiple sources, which may include previously reported experiments, computational predictions and other database-derived evidence. Evidence types are not distinguished in this figure, and no interactions were experimentally validated in the present study. Neither the network edges nor hub status establish that the represented proteins interact directly with MATR3. Abbreviations: MATR3, matrin-3; mRNA, messenger RNA; STRING, Search Tool for the Retrieval of Interacting Genes/Proteins.

STRING-derived protein association networks were constructed separately for the proteins encoded by the 50 genes most strongly positively correlated and the 50 genes most strongly negatively correlated with *MATR3* mRNA expression, and visualised in Cytoscape v3.10.3. These gene sets were selected by the strength and direction of their expression correlations, not by evidence of interaction with the MATR3 protein. Both networks showed significant PPI enrichment (nominal *p* < 1.0 × 10^−16^), indicating more associations among the selected proteins than expected under the STRING enrichment model (Figure 8). This enrichment does not establish physical interactions or functional relationships between these proteins and MATR3 itself.

For the genes positively correlated with *MATR3* mRNA expression (Figure 8A,B), the corresponding PPI network comprises 50 nodes and 259 edges, with an average node degree of 10.36, a clustering coefficient of 0.439, a network density of 0.211, a diameter of 3, and a characteristic path length of 1.985. Among the ten most connected hub proteins, SMARCA5, CDC5L, and NIPBL have the highest node degrees. For the genes negatively correlated with *MATR3* mRNA expression (Figure 8C,D), the corresponding PPI network comprises 50 nodes and 382 edges, with a higher average node degree (15.28), a clustering coefficient of 0.642, a network density of 0.312, a diameter of 4, and a characteristic path length of 1.858, indicating a denser network of protein associations. The hub proteins with the highest node degrees are NDUFS8, NDUFB7, and NDUFA13.

## 3. Discussion

Understanding the mechanisms of action of MATR3 in various biological contexts may provide the basis for precisely determining the role of this protein in the processes of carcinogenesis. Its complex role in the cell is the subject of intensive scientific research, but the complexity and dynamics of the processes occurring in various types of cancer make it difficult to determine its exact function. One of the cancers characterised by high biological and clinical complexity is endometrial carcinoma (EC); therefore, identifying a reliable prognostic marker for this malignancy represents an important scientific objective. Among many potential markers, the MATR3 protein stands out, as its features may act as a prognostic indicator for EC. Many functions are attributed to it, such as regulation of chromosome and genome integrity through interaction with DNA/RNA-binding proteins; binding to adenine/thymine-rich DNA sequences called S/MAR, which affects chromatin structure; and participation in DNA repair processes by accumulating and stabilising mRNA encoding key factors such as RAD51 [12,23,24]. So far, the mechanisms of action of MATR3 involved in carcinogenesis in the context of EC have not been described.

### 3.1. MATR3 Expression in Endometrial Carcinoma and Comparison with Other Tumour Types

The present study characterises MATR3 expression and its association with clinical outcomes in EC. MATR3 protein expression was lower in EC tissues than in normal endometrium (*p* < 0.0001), and lower *MATR3* mRNA expression was also observed in the transcriptomic comparison. These observations establish differences in expression but do not demonstrate a tumour-suppressive function or a causal role in disease progression. Although the physiological role of MATR3 has not been specifically characterised in normal endometrial tissue, its higher expression in normal endometrium in our cohort is consistent with its established cellular functions in RNA metabolism. MATR3 has been shown to bind and stabilise selected mRNAs and to regulate alternative splicing, while more recent studies demonstrated that its loss promotes cryptic exon inclusion in multiple transcripts [16,17,18]. Together with the previously reported roles of MATR3 in chromatin organisation and stem cell maintenance [23,24,25], these findings indicate that MATR3 participates in several processes required for normal cellular function. However, whether these mechanisms contribute specifically to endometrial homeostasis remains unknown.

The lower MATR3 protein expression observed in our institutional EC cohort is consistent with findings reported for basal-like breast cancer (BLBC), where *MATR3* was identified as one of the genes deleted from chromosome 5, suggesting a tumour-suppressive function [21]. In BLBC, forced overexpression of the MATR3 protein inhibited tumorigenicity and cell migration, further supporting its suppressor activity. Similarly, lower *MATR3* mRNA levels in tumour tissues relative to controls have been reported in clear cell renal cell carcinoma (ccRCC), where low expression independently predicted poor overall survival [26].

In contrast, the present results introduce an important dichotomy relative to tumour types such as hepatocellular carcinoma (HCC) and malignant melanoma (MM), where *MATR3* is described as an oncogene with elevated levels correlated with poor prognosis. In HCC, overexpression of *MATR3* mRNA was associated with unfavourable overall survival [27], and a subsequent study demonstrated that patients with high MATR3 levels exhibited significantly shorter overall, disease-free, progression-free, and disease-specific survival [28]; the same work confirmed that MATR3 promotes liver cancer progression by suppressing DHX58-mediated type I interferon response. In the present study of EC, by contrast, high *MATR3* mRNA expression correlates with improved overall survival (median OS = 110.1 months), underscoring the tissue-specific nature of this protein’s function. Furthermore, Yang et al. [14] showed that high *MATR3* mRNA expression in neuroblastoma was significantly associated with worse event-free and overall survival, further illustrating the context-dependent nature of MATR3 biology across cancer types.

An interesting reference point is non-small cell lung cancer (NSCLC), where a discrepancy between mRNA and protein levels was observed: *MATR3* mRNA was elevated in tumour tissue compared with controls, yet reduction in MATR3 protein expression proved to be an independent unfavourable prognostic factor [29]. In the present EC study, lower MATR3 expression was observed at both the protein and mRNA levels in separate cohorts, but the mechanisms underlying these expression differences were not investigated. Collectively, these findings highlight marked tissue-specific differences in MATR3 expression and underscore its context-dependent role in tumour biology.

### 3.2. Prognostic Relevance of MATR3 in Endometrial Carcinoma

The prognostic relevance of MATR3 expression was clearly demonstrated in the present cohort, where high MATR3 levels were significantly associated with improved overall survival in EC patients. Kaplan–Meier survival analysis revealed that patients with high *MATR3* mRNA expression had a median OS of 110.1 months, whereas individuals with reduced *MATR3* expression had more than twice the risk of death (HR = 2.174; 95% CI: 1.066–4.43), underscoring the potential utility of MATR3 as a prognostic biomarker in endometrial carcinoma. These data are fully consistent with findings in ccRCC, where low MATR3 expression independently predicted poor overall survival [26]. Consistent with the transcriptomic findings, low MATR3 protein expression in our immunohistochemical cohort was also associated with significantly shorter overall survival and remained an independent prognostic factor in multivariable Cox regression analysis.

In the TCGA cohort, high *MATR3* mRNA expression was identified in 97 of 172 EC cases (56.4%). No statistically significant associations with age, histological grade, or histological type were observed before or after Benjamini–Hochberg adjustment across the three clinicopathological comparisons (adjusted *p* = 0.8991 for each; Table 2 and Table S1). A comparable proportion was reported in a TCGA-based ccRCC study (276 of 475 patients; 58.1%), where most clinicopathological parameters were similarly not significantly related to expression levels; however, in ccRCC, a significant association with age was noted—a relationship not observed in the present EC cohort. It is worth noting that the prognostic role of MATR3 is strictly dependent on cancer type: while in EC and ccRCC low expression constitutes an unfavourable prognostic factor, in HCC and neuroblastoma, it is precisely high MATR3 levels that correlate with worse clinical outcomes, reinforcing the tissue-specific character of this biomarker.

In the institutional IHC cohort, low MATR3 expression was more frequent in tumours with advanced pT status and lymph-node involvement, and an association with FIGO stage was also observed at the nominal significance level. However, none of these associations remained statistically significant after Benjamini–Hochberg adjustment across the nine clinicopathological comparisons (adjusted *p* = 0.0885 for each; Supplementary Table S1). These findings should therefore be interpreted as exploratory. The absence of significance after multiplicity adjustment does not exclude potential confounding by established prognostic factors. In the adjusted survival analysis, the association between low MATR3 protein expression and shorter OS persisted after adjustment for age, histological grade, pT, pN, and pM and stratification by LVSI status. Furthermore, adding MATR3 to the corresponding clinical model improved relative model fit and produced a modest numerical increase in apparent discrimination (Supplementary Table S4). These findings suggest that the prognostic association of MATR3 is not fully explained by the clinicopathological factors included in the model. However, adjustment cannot exclude residual confounding, and improved model fit does not establish clinical utility. Independent validation is required to determine whether MATR3 improves risk assessment beyond conventional pathological parameters in other patient populations.

The molecular heterogeneity of EC provides a rationale for investigating the prognostic association of *MATR3* within individual TCGA subtypes. In our exploratory analyses, higher *MATR3* mRNA expression was associated with improved OS in the CN-low subgroup at the nominal significance level; however, this association did not remain significant after Benjamini–Hochberg adjustment (adjusted *p* = 0.0983) and was based on only two deaths. The MSI-H and CN-high comparisons were not statistically significant, while no deaths occurred in the POLE subgroup, precluding an informative comparison of survival between expression groups. These findings do not establish that the prognostic association of *MATR3* differs across molecular subtypes. The distinct biological characteristics of these subgroups, including the extensive copy-number alterations and frequent TP53 mutations characteristic of CN-high tumours, remain relevant to the investigation of potential biomarkers, but their influence on the association between *MATR3* and survival was not directly tested in this study. The nominal finding in CN-low tumours may motivate further investigation, but it does not establish additional risk-stratification value within this molecular category. Larger independent cohorts with sufficient events and formal tests of interaction between *MATR3* expression and molecular subtype are needed to evaluate possible subtype-specific prognostic associations.

The exploratory methylation analysis identified weak negative correlations between *MATR3* mRNA expression and methylation at two of the 34 examined gene-body CpG probes in TCGA-UCEC. Only one of these probes, cg04447756, also showed a statistically significant association in the additional MEXPRESS analysis. Because the analyses used overlapping TCGA data, this finding does not constitute independent validation. These limited, site-specific associations do not establish DNA methylation as a mechanism responsible for reduced *MATR3* expression in EC. Whether methylation at these sites influences *MATR3* expression remains unresolved and requires functional investigation. Both CpG sites are intronic relative to *MATR3* transcript ENST00000394805. Intragenic methylation has been implicated in the regulation of alternative promoter usage and pre-mRNA splicing in other experimental systems [30,31]. This provides a biological rationale for investigating these sites, but their genomic location alone does not establish a regulatory function. Our analyses did not assess transcript-specific expression, promoter activity, or enhancer activity, and therefore cannot determine whether methylation at these sites affects *MATR3* transcription or RNA processing.

### 3.3. Molecular Context: Cell Cycle, Apoptosis, and Known MATR3 Interactions

In almost all cancers, disruptions in cell cycle regulation lead to uncontrolled cellular proliferation, encouraging the identification of targeted cell cycle regulators whose understanding is crucial for prognosis and therapy development [32]. In vitro studies have revealed that MATR3 promotes binding of the lncRNA *PINCR* to the enhancer region of p53-inducing genes, thereby reducing the sensitivity of colorectal cancer cells to DNA damage; matrin-3 and *PINCR* together regulate the induction of a subset of p53 targets involved in G1 cell arrest and apoptosis [33]. Moreover, inhibition of MATR3 expression induces apoptosis in oral squamous cell carcinoma cells, as evidenced by increased levels of cleaved PARP and caspase-3 in siMATR3-transfected cells [34].

A similar pro-apoptotic relationship was observed in malignant melanoma, where MATR3 knockdown resulted in both inhibition of proliferation and activation of apoptosis; MATR3 has been shown to be associated with the FGF2 signalling pathway, promoting cell cycle progression by regulating the expression of AKT, p38, and p21 [22]. Conversely, according to more recent reports, MATR3 acts as a suppressor in basal-like breast cancer by suppressing genes responsible for the activation of the oncogenic YAP/TAZ pathway and by promoting apoptotic death of cancer cells [21]. These discrepancies highlight the context-dependent role of MATR3 across different tumour types and underscore the need for further mechanistic studies.

### 3.4. Co-Expression Patterns and Functional Annotations Associated with MATR3

The co-expression analysis revealed both positive and negative relationships between *MATR3* mRNA abundance and the transcript levels of other genes in EC. Based on Spearman’s rank correlation coefficients, *BCLAF1* and *TOP2B* showed the strongest positive associations with *MATR3* mRNA expression, whereas *TRAPPC5* and *FASTK* showed the strongest inverse associations. The MATR3 protein contributes to gene expression regulation through several mechanisms, including stabilisation of target RNAs and modulation of chromatin architecture that supports transcription factor activity [23]. The correlations with *BCLAF1* and *TOP2B* identify candidates for further investigation but do not establish direct interactions or a shared tumour-suppressive mechanism.

*BCLAF1* (BCL-2-associated transcription factor 1) has been implicated in the regulation of transcription, apoptosis, and mRNA splicing, and has been linked to tumour suppressor functions in several malignancies. The positive correlation between *MATR3* and *BCLAF1* expression (ρ = 0.785) is a co-expression finding and does not establish an effect of *MATR3* on splicing or apoptosis in EC. *TOP2B* (DNA topoisomerase II beta) plays a critical role in DNA replication and transcriptional regulation, and its dysregulation has been associated with genomic instability and tumorigenesis. Similarly, the correlation with *TOP2B* (ρ = 0.745) does not demonstrate that *MATR3* regulates chromatin architecture or the DNA damage response in the analysed tumours [25].

In contrast, *FASTK* (Fas-activated serine/threonine kinase), which exhibited one of the strongest negative correlations with *MATR3* (ρ = −0.687), is known to regulate mitochondrial RNA processing and has been implicated in mitochondrial function and apoptosis [35]. *TRAPPC5*, a component of the transport protein particle (TRAPP) complex involved in vesicle-mediated transport and membrane trafficking, also showed a pronounced negative correlation with *MATR3* [36,37]. The inverse correlations with *FASTK* and *TRAPPC5* identify relationships between transcript levels but do not demonstrate suppression of mitochondrial or vesicular transport processes by *MATR3*.

After correction for multiple testing, the enrichment analyses supported RNA-related pathway annotations and nuclear cellular components among genes positively correlated with *MATR3* mRNA expression, together with mitochondrial pathway annotations and cellular components among negatively correlated genes. However, several terms that reached nominal significance did not remain significant after adjustment, including mitotic cytokinesis in the positively correlated set and aerobic respiration and protein binding in the negatively correlated set in the GO analysis. These findings describe the functional annotations of the selected gene sets and do not demonstrate opposing pathway activities or a causal regulatory role for MATR3.

The genes identified by our co-expression analysis should be distinguished from experimentally demonstrated MATR3 interaction partners. Here, expression correlation denotes an association among transcript abundances across tumour samples, whereas STRING edges represent database-derived protein associations supported by different types of evidence and do not necessarily indicate direct physical binding. In the STRING-derived networks, highly connected nodes included SMARCA5, CDC5L and NIPBL in the positively correlated set, and NDUFS8, NDUFB7 and NDUFA13 in the negatively correlated set. Their hub status describes connectivity within the selected networks; it does not identify them as MATR3-binding proteins or MATR3-regulated targets. The broader term “MATR3-associated” should therefore not be interpreted as evidence of molecular interaction. Experimentally supported interactions involving MATR3 discussed elsewhere in this manuscript derive from the cited studies and their specific experimental models, not from the present co-expression or STRING analyses. No MATR3 interaction partners were experimentally validated in the present study.

### 3.5. Biological Hypotheses and Directions for Functional Validation

The enrichment of nuclear transcription and RNA-processing annotations among genes positively correlated with *MATR3* mRNA expression, and mitochondrial annotations among negatively correlated genes, describes patterns of co-expression in bulk tumour transcriptomes. Such patterns may be influenced by differences in tumour purity and the proportions of malignant, stromal and immune cells [38]. Differences in proliferative activity and other tumour characteristics may also contribute to the observed correlations. The mitochondrial signature could therefore partly reflect variation in cellular composition rather than a direct biological effect of MATR3 protein within tumour cells. Because these factors were not accounted for in the co-expression analysis, their contribution cannot be distinguished from a potential *MATR3*-related association. These findings do not demonstrate increased mitochondrial biogenesis, oxidative phosphorylation activity, or metabolic reprogramming caused by reduced *MATR3* mRNA expression. Similarly, the PPI analysis provides a network context for the selected genes but does not establish MATR3-dependent interactions in endometrial carcinoma. Analyses accounting for tumour purity and cellular composition, together with functional experiments, would be needed to determine whether these associations have a mechanistic basis.

Previous experiments in non-endometrial cell models provide a rationale for investigating RNA-processing mechanisms in EC. MATR3 depletion has been reported to alter alternative splicing and promote cryptic exon inclusion, with some resulting transcripts predicted or experimentally shown to undergo nonsense-mediated decay [17,18]. Whether comparable effects occur in EC remains unknown. Functional studies manipulating MATR3 expression and directly measuring RNA processing and mitochondrial phenotypes are needed to determine whether the observed co-expression patterns have a mechanistic basis. Previous experimental studies have identified different roles for MATR3 in other tumour types. In HCC, MATR3 was reported to promote tumour progression through suppression of the DHX58-mediated type I interferon response [28], whereas experimental findings in basal-like breast cancer supported a tumour-suppressive role [21]. These observations provide a rationale for investigating the biological function of MATR3 in EC but do not establish a corresponding mechanism in this tumour type. In our study, higher MATR3 protein expression in the institutional cohort and higher *MATR3* mRNA expression in the TCGA cohort were associated with more favourable survival, while the mechanisms underlying these associations remain to be determined.

The correlations of *MATR3* mRNA expression with *TOP2B* and *BCLAF1* provide a rationale for investigating a possible relationship with DNA damage responses in EC. They do not establish a role for the MATR3 protein in genome maintenance in this tumour type [39].

Furthermore, the literature from other cancer models indicates that MATR3 participates in the early response to double-strand DNA breaks (DSBs): in malignant melanoma, MATR3 has been shown to interact directly with ATM kinase (a key DNA damage sensor) in response to genotoxic stress [22,40], while in HCC it was identified as a partner of RAD21, a protein involved in DSB repair [27]. MATR3 also forms complexes with SFPQ and NONO, which participate in nuclear retention and the DNA damage response [40]. These findings from other experimental systems motivate further investigation, but the present study does not establish that loss of MATR3 impairs DNA repair or drives tumour progression in EC.

### 3.6. Study Limitations

This study has several limitations. Its retrospective, single-centre design and relatively small institutional immunohistochemical (IHC) cohort of 112 patients may introduce selection bias and limit the generalisability of the findings. Although 60 deaths were recorded in this cohort, some clinicopathological subgroups contained few patients and events, limiting the precision of the estimates. The absence of statistically significant associations for pT, pN and pM should therefore not be interpreted as evidence against their established prognostic relevance.

The immunohistochemical assessment was subject to tissue sampling limitations. Each tumour was represented by one tissue fragment in a macroarray, and MATR3 protein expression was assessed in three tumour-containing microscopic fields. This approach did not capture spatial variation across different tumour regions, and the resulting H-score may not fully reflect intratumoral heterogeneity in MATR3 protein expression. The control tissues were obtained from patients undergoing hysterectomy for uterine fibroids and predominantly represented postmenopausal, atrophic endometrium. Although older patients were preferentially selected to improve age comparability, no formal age matching was performed. Differences in age, menopausal status and clinical context may therefore have influenced the tumour–control comparison, and the observed difference in MATR3 protein expression cannot be attributed solely to malignant transformation.

The survival analyses were limited by the availability of clinical information. None of the patients in the institutional cohort received radiotherapy, chemotherapy or hormonal treatment before surgery, but data on postoperative exposure to these treatments were unavailable. Treatment differences could therefore influence overall survival independently of MATR3 protein expression and could not be accounted for in the analyses. Adjustment was restricted to the clinicopathological factors and categorical definitions included in the models, leaving the possibility of residual confounding. Data on disease recurrence, progression, response to subsequent treatment and cause of death were also unavailable. Consequently, these outcomes could not be evaluated, and deaths attributable to endometrial carcinoma could not be distinguished from deaths due to other causes.

The cut-offs for MATR3 protein expression and *MATR3* mRNA expression were selected using survival outcomes, potentially leading to optimistic estimates of prognostic associations. Subsequent Cox inference and model comparisons did not account for the original threshold search. Revisions to the institutional Cox models were undertaken during peer review, and model discrimination was evaluated in the same cohort used for model fitting without correction for optimism. The model comparisons should therefore be considered exploratory. The numerical increase in apparent Harrell’s C does not establish improved prediction in new patients; calibration and clinical net benefit were not evaluated. Stratification accommodated evidence of non-proportional hazards for lymphovascular space invasion but precluded estimation of a single adjusted hazard ratio for this variable. In addition, the study involved multiple statistical comparisons, increasing the risk of chance findings. Multiplicity adjustment was applied within specified analysis families but did not cover all exploratory analyses or outcome-based cut-off selection. Nominal associations should therefore be interpreted cautiously.

The TCGA survival analysis included 172 patients and 31 deaths. Event counts were particularly limited within molecular subtypes, with only two deaths in the CN-low subgroup and none in the POLE subgroup. These small event counts restrict the precision and interpretability of subtype-specific findings. The exploratory subgroup analyses do not establish that the prognostic association of *MATR3* mRNA expression is independent of molecular subtype or differs between subtypes, and residual confounding by subtype cannot be excluded.

The institutional IHC and TCGA transcriptomic analyses involved different patients and measurement platforms. The TCGA findings provide complementary evidence at the mRNA level but do not directly validate the IHC assay, its H-score cut-off or the institutional clinical model. No independent IHC cohort was available for external validation. Furthermore, transcript abundance does not necessarily correspond to protein abundance because of post-transcriptional regulation, translational control and protein turnover. Limitations in the available archival formalin-fixed paraffin-embedded material precluded additional mRNA analysis in the institutional cohort. Paired measurements of *MATR3* mRNA and MATR3 protein were therefore unavailable, preventing assessment of their correlation within the same patients. Independent validation using standardised protein assessment and prospectively specified analyses is required.

The methylation, co-expression, pathway enrichment and STRING-derived protein association analyses are hypothesis-generating. The bulk transcriptomic co-expression analysis was not adjusted for tumour purity, stromal or immune cell contributions, or proliferative activity. The observed correlations with *MATR3* mRNA expression and the associated functional enrichments may therefore partly reflect cellular composition or other confounding factors rather than MATR3-dependent processes within malignant cells. Enrichment results also depend on the selected gene lists, database annotations and reference backgrounds; the use of fixed-size correlation-based gene lists and the tools’ default reference backgrounds should be considered when interpreting these findings.

Finally, correlations and database-derived networks do not establish causal regulation, pathway activity or direct molecular interactions in endometrial carcinoma. No functional or mechanistic experiments were performed. The present findings therefore do not establish a tumour-suppressive function of MATR3, a causal role in tumour progression or its suitability as a therapeutic target.

## 4. Materials and Methods

### 4.1. Patient Cohort and Tissue Specimens

Formalin-fixed paraffin-embedded (FFPE) specimens from 112 patients with EC who underwent total hysterectomy with pelvic lymphadenectomy at the Department of Obstetrics, Gynaecology and Oncology, Dr. Jan Biziel Memorial University Hospital No. 2 in Bydgoszcz, Poland, between 2012 and 2021, were included in the study. None of the patients received radiotherapy, chemotherapy, or hormonal treatment before surgery. Two pathologists independently reviewed the tumour specimens to verify the histopathological diagnoses. Tumour staging was reassessed according to the seventh edition of the American Joint Committee on Cancer (AJCC) TNM classification. This was a retrospective study based on archival FFPE material. The sample size was determined by the number of eligible cases available in the institutional archive during the defined study period. All consecutive cases meeting the inclusion criteria, including sufficient representative tumour tissue, complete clinicopathological data, and available follow-up information, were included. The control group consisted of endometrial tissue samples obtained from 31 patients who underwent hysterectomy for histologically confirmed uterine fibroids at the same department. No other concomitant gynaecological conditions were documented, and none of the patients had received hormonal therapy before surgery. Older patients were preferentially selected to improve age comparability with the endometrial carcinoma cohort; however, no formal age-matching procedure was applied. The median age was 59 years (interquartile range, 55–65 years; range, 45–69 years). Twenty-seven patients (87.1%) were postmenopausal, and four (12.9%) were menstruating. Histopathological examination showed atrophic endometrium in 27 cases (87.1%), proliferative endometrium in two cases (6.5%), and secretory endometrium in two cases (6.5%), with no evidence of hyperplasia, atypia, significant inflammation, or neoplastic changes.

The study cohort included 37 patients aged ≤60 years and 75 patients aged >60 years. Most tumours were classified as histological grade G2 or G3, with 7 G1, 63 G2, and 42 G3 cases. Type I endometrial carcinoma was diagnosed in 89 patients, whereas type II tumours were identified in 23 patients. According to tumour stage, 93 cases were classified as pT1–T2 and 19 as pT3–T4. Lymph node metastases were present in 20 patients, and distant metastases were observed in 11 patients. Lymphovascular space invasion was identified in 22 cases. The FIGO distribution was as follows: stage I, 51 cases; stage II, 28 cases; stage III, 23 cases; and stage IV, 10 cases. Myometrial invasion ≥ 50% was observed in 78 patients. The detailed characterization of the study group is shown in Supplementary Table S6.

The date of 13 April 2022 was set as the cut-off for OS analysis. OS was defined as the time from diagnosis to the last follow-up or death.

This study was approved by the Ethics Committee of Collegium Medicum in Bydgoszcz, Nicolaus Copernicus University in Torun (number KB 87/2020; date of approval: 25 February 2020).

### 4.2. Immunohistochemistry

Tissue macroarrays were assembled and prepared for immunohistochemical staining following the protocol reported by Antosik et al. [41]. For each patient, one representative tissue fragment containing carcinoma and associated stroma was selected from the hysterectomy material for tissue macroarray construction. The fragments measured approximately 1.0–1.2 × 0.8–1.0 × 0.5 cm. Each recipient macroarray block contained up to five fragments, each originating from a different patient. Representative regions with a tumour cell content of at least 80% were selected for tissue macroarray construction. The resulting paraffin blocks were sectioned at a thickness of 4.0 μm using an Accu-Cut manual rotary microtome (Sakura, Torrance, CA, USA). Sections encompassing the selected tumour regions were transferred onto Superfrost Plus high-adhesion slides (Menzel Gläser, Germany).

Immunohistochemical staining was performed using the BenchMark ULTRA automated slide processing system (Roche Diagnostics/Ventana Medical Systems, Tucson, AZ, USA). The automated staining workflow followed the manufacturer’s instructions and included deparaffinisation, antigen retrieval, antibody incubation, signal detection and counterstaining. Antigens were retrieved by treatment with high-pH Cell Conditioning 1 buffer for 64 min. MATR3 protein was labelled with a rabbit polyclonal anti-MATR3 antibody (cat. no. HPA036565; Sigma-Aldrich, St. Louis, MO, USA), applied at a 1:300 dilution for 32 min. Immunoreactivity was visualised with the Ventana ultraView Universal DAB Detection Kit (Ventana Medical Systems, Inc., Tucson, AZ, USA) using 3,3′-diaminobenzidine as the chromogenic substrate. Following haematoxylin counterstaining, the sections were dehydrated and mounted under coverslips.

The antibody has been characterized for immunohistochemical use in FFPE tissues, with its IHC performance rated as “Supported” by the Human Protein Atlas. Its use in FFPE tumour specimens has also been reported previously [26]. Appropriate positive and negative controls were included in each staining run to validate staining specificity. Human colon tissue served as a positive control for MATR3 immunostaining, showing strong nuclear staining in glandular epithelial cells. Negative controls were prepared by omitting the primary antibody.

### 4.3. Expression Analysis

MATR3 immunostaining was evaluated using slides digitised with the Roche Ventana DP 200 slide scanner (Ventana Medical Systems, Inc., Tucson, AZ, USA). Two independent assessments were performed for each case: one by a team comprising J.P., E.P., and N.S.-M., and the other by D.G. All evaluators were blinded to patient outcome data. Each assessment was based on three tumour-containing microscopic fields evaluated at ×20 magnification, avoiding necrotic areas and inflammatory cell-rich regions. Only nuclear staining in tumour cells was scored.

MATR3 expression was quantified using the H-score, calculated as the sum of the percentages of tumour cells showing weak, moderate, and strong staining multiplied by their respective intensity scores: H-score = (1 × % weakly stained cells) + (2 × % moderately stained cells) + (3 × % strongly stained cells), yielding a score ranging from 0 to 300. The two independently recorded H-scores were averaged to obtain the final score for each case. The final H-score was used to classify cases into low- and high-expression groups using a survival-based cut-off determined with Evaluate Cutpoints software (version 1.0) and the cutp method implemented in the survMisc package (version 0.5.6) in R (version 4.4.3). Candidate cut-off values were evaluated using the absolute log-rank score for overall survival, and the value yielding the maximum score was selected. The resulting cut-off was 158; cases with H-scores < 158 were classified as having low MATR3 expression (*n* = 62), whereas those with H-scores ≥ 158 were classified as having high MATR3 expression (*n* = 50).

### 4.4. In Silico Transcriptomic Analysis

*MATR3* mRNA expression values for the 172-sample TCGA-UCEC analytical cohort and normal non-tumour tissue samples from the Genotype-Tissue Expression (GTEx) project were obtained from the UCSC Xena Toil hub (https://toil.xenahubs.net; accessed 26 August 2026), dataset TcgaTargetGtex_RSEM_Hugo_norm_count (version 2016-04-12). The analysed gene was *MATR3* (Ensembl identifier: ENSG00000280987.2), with expression values retrieved from the row labelled *MATR3* in this gene-symbol-based dataset. Values were supplied as log2(norm_count + 1) and were used on this scale without additional transformation. Detailed characteristics of the TCGA analytical cohort are presented in Supplementary Table S7. This cohort was used as an independent exploratory transcriptomic cohort providing complementary evidence at the mRNA level, rather than as an external validation dataset for the institutional IHC findings. *MATR3* expression and overall survival data retrieved from UCSC Xena were integrated with clinical information from the TCGA PanCancer Atlas UCEC study in cBioPortal using TCGA sample identifiers. Cases lacking overall survival time or vital status, or without a matching sample identifier in the selected cBioPortal dataset, were excluded during dataset assembly. The resulting analytical cohort comprised 172 primary endometrial carcinoma samples with available *MATR3* mRNA expression and complete overall survival information. Missing annotations for individual downstream analyses were handled separately.

All 172 cases in the analytical cohort had recorded overall survival time and vital status and were included in the survival analysis (31 deaths and 141 censored observations). The survival-based *MATR3* cut-off was determined using Evaluate Cutpoints and the cutp method in survMisc. The existing threshold of 10.17 was retained: values < 10.17 defined low expression (*n* = 75), and values ≥ 10.17 defined high expression (*n* = 97). The same threshold was applied within each molecular subtype without subtype-specific optimisation. Exploratory subtype-specific hazard ratios comparing low with high MATR3 expression were estimated using univariable Cox proportional hazards models with Efron handling of tied event times. Hazard ratios and 95% Wald confidence intervals were reported where finite estimates could be obtained. Six cases without molecular subtype annotation were omitted only from subtype-specific analyses, leaving 166 cases: CN-high, *n* = 82; MSI-H, *n* = 39; CN-low, *n* = 30; and POLE, *n* = 15.

DNA methylation beta values were obtained from the UCSC Xena TCGA hub (https://tcga.xenahubs.net), accessed 26 August 2026, dataset TCGA.UCEC.sampleMap/HumanMethylation450 (dataset release dated 8 September 2017). CpG probes mapped to *MATR3* in the UCSC Xena probe annotation were selected for further analysis. In total, 34 CpG probes were included.

DNA methylation data were merged with *MATR3* mRNA expression data by TCGA sample barcode. Four samples with missing methylation values were excluded, resulting in 168 cases available for correlation analysis. To assess the reproducibility of significant findings, CpG probes identified in the primary correlation analysis were additionally evaluated using the MEXPRESS platform in a larger publicly available TCGA-UCEC cohort of primary tumour samples. Multiple testing correction was performed using the Benjamini–Hochberg false discovery rate method as implemented in MEXPRESS.

Co-expression analysis of *MATR3* mRNA expression (Entrez Gene ID: 9782) was performed using the TCGA PanCancer Atlas UCEC study in cBioPortal (ucec_tcga_pan_can_atlas_2018) and the RNASeqV2 expression profile ucec_tcga_pan_can_atlas_2018_rna_seq_v2_mrna. This analysis comprised 527 cases with available expression data and was distinct from the 172-case survival cohort. Spearman’s rank correlations were calculated between *MATR3* mRNA expression and the expression of other genes. The 50 most positively and 50 most negatively correlated genes with *MATR3* mRNA expression were selected for downstream exploratory enrichment and network analyses. Gene selection was based on the strength and direction of Spearman’s correlation coefficients rather than a statistical significance threshold. The gene-level *p*-values reported in Table 4 and Table 5 are nominal and are presented for descriptive purposes; they should not be interpreted as evidence of significance after correction for genome-wide multiple testing. These fixed-size gene sets were used to generate hypotheses through downstream enrichment and network analyses.

Functional enrichment analyses were performed separately for the 50 genes most positively and the 50 genes most negatively correlated with *MATR3* mRNA expression. Reactome pathway and Gene Ontology (GO) enrichment analyses were repeated on 19 October 2026 using Reactome release 97 and the Database for Annotation, Visualization and Integrated Discovery (DAVID; Knowledgebase v2026_2), respectively.

Reactome over-representation analysis used human projection and the default reference background, with disease pathways retained and no additional interactors included. Statistical significance was assessed using the Benjamini–Hochberg-adjusted *p*-values returned by Reactome. GO enrichment was evaluated in DAVID across biological process, cellular component and molecular function categories using GOTERM_BP_DIRECT, GOTERM_CC_DIRECT and GOTERM_MF_DIRECT, respectively. Analyses used the default Homo sapiens background and the EASE score (modified Fisher exact test). Benjamini–Hochberg adjustment was applied separately within each GO category and gene set. All returned GO terms were retained for reporting, including terms with a single mapped gene (count threshold 1; EASE threshold 1). An adjusted *p*-value < 0.05 defined significant enrichment. Selected results, including nominal and adjusted *p*-values, are presented in Supplementary Table S5.

Protein–protein interaction networks were generated using the STRING database (accessed 8 August 2025) with a minimum interaction score of 0.700. STRING network-enrichment *p*-values were reported as nominal values without adjustment for multiple testing. The resulting networks were visualized in Cytoscape version 3.10.3, and the cytoHubba plugin was used to identify hub nodes within networks constructed from genes correlated with *MATR3* mRNA expression. Network connectivity and hub rankings were interpreted as descriptive properties of database-derived networks. Both enrichment and network analyses were considered exploratory and do not establish pathway activation, direct molecular interactions in EC, or causal regulation by the MATR3 protein.

### 4.5. Statistical Analysis

Statistical analyses were performed using GraphPad Prism version 11 and R version 4.4.3. The normality of continuous variable distributions was assessed using the Shapiro–Wilk test. Categorical variables were summarised as counts and percentages, with denominators specified in the corresponding tables. Associations between expression categories and clinicopathological characteristics were evaluated using the chi-square test or Fisher’s exact test, as appropriate. Differences in MATR3 protein expression between carcinoma and control tissues, differences in *MATR3* mRNA expression between tumour and non-tumour tissues, and comparisons between histological types were assessed using two-sided Mann–Whitney U tests. *MATR3* mRNA expression across the four TCGA molecular subtypes was compared using the Kruskal–Wallis test.

Agreement between the two independently recorded H-scores was evaluated using a two-way mixed-effects intraclass correlation coefficient with an absolute-agreement definition. Reliability was assessed for a single assessment using ICC(A,1) and for the mean of the two assessments using ICC(A,2). The corresponding 95% confidence intervals were estimated using a percentile bootstrap with 50,000 resamples, resampling patients while retaining their paired assessments.

Overall survival (OS) was analysed in months, with death from any cause treated as the event and surviving patients censored at their last recorded follow-up. In the institutional cohort, OS was measured from diagnosis, with follow-up closed on 13 April 2022. In the TCGA cohort, the recorded OS times and vital status were used. Survival distributions were estimated using the Kaplan–Meier method and compared using the log-rank (Mantel–Cox) test. Analyses distinguished MATR3 protein expression in the institutional cohort from *MATR3* mRNA expression in the TCGA cohort. The survival-derived thresholds described in Section 4.3 and Section 4.4 were retained: H-score < 158 versus ≥158 for protein expression and expression values < 10.17 versus ≥10.17 for mRNA expression. These thresholds were not re-optimised during the revised survival analyses or within molecular subtypes. The overall-cohort log-rank-derived hazard ratios and their 95% confidence intervals were distinguished from estimates obtained using Cox regression.

Cox regression analyses of the institutional cohort included all 112 patients, with 60 deaths and complete data for the model covariates. Tied event times were handled using Efron’s method. Univariable models evaluated MATR3 protein expression (low versus high), age (>60 versus ≤60 years), histological grade (G3 versus G1–G2), pT (T3–T4 versus T1–T2), pN (N1 versus N0), pM (M1 versus M0), and lymphovascular space invasion (LVSI; present versus absent). Hazard ratios (HRs), 95% confidence intervals (CIs), and two-sided Wald *p*-values were reported.

The proportional hazards assumption was examined using scaled Schoenfeld residual plots and tests with a Kaplan–Meier transformation of time, supplemented by log-minus-log survival plots. Evidence of non-proportional hazards for LVSI prompted the use of an LVSI-stratified Cox model as the revised main adjusted analysis. This model included MATR3 protein expression, age, histological grade, pT, pN, and pM simultaneously, retaining all six covariates irrespective of statistical significance. Separate baseline hazards were allowed for patients with and without LVSI; consequently, no common adjusted HR was estimated for LVSI.

Sensitivity analyses comprised the full unstratified seven-variable model, a four-variable model including MATR3 protein expression, age, histological grade, and LVSI, and an LVSI-stratified model including MATR3 protein expression, age, and histological grade. A leave-one-out analysis assessed the influence of individual patients on the MATR3 coefficient in the revised main model. To examine the previously reported reduced model, exploratory backward elimination was repeated from the seven-variable model, sequentially removing the covariate with the highest Wald *p*-value when *p* > 0.10. A separate analysis starting with the four covariates significant in univariable analyses yielded the same reduced model. These selection procedures were not used to determine the covariates retained in the revised main model.

The incremental prognostic information associated with MATR3 protein expression was explored by comparing two nested LVSI-stratified Cox models fitted to the same 112 patients. The clinical model included age, histological grade, pT, pN, and pM; the extended model additionally included MATR3 protein expression. Model fit was compared using a partial likelihood-ratio test with one degree of freedom and the Akaike information criterion (AIC). Apparent discrimination was assessed using Harrell’s concordance index (C), calculated from comparable patient pairs within LVSI strata and pooled across strata. Changes in C were reported descriptively without a formal significance test.

The TCGA survival analysis included 172 patients and 31 deaths. Analyses requiring molecular subtype annotations included 166 patients; the six patients without these annotations were excluded only from the corresponding analyses. Subtype-specific survival comparisons used the same mRNA expression threshold as the overall TCGA analysis. Exploratory subtype-specific HRs comparing low with high *MATR3* mRNA expression were estimated using univariable Cox models with Efron handling of tied event times and 95% Wald CIs where finite estimates could be obtained. Neither a log-rank *p*-value nor an HR could be estimated for the POLE subgroup because no deaths occurred. In the MSI-H and CN-low subgroups, no finite maximum partial-likelihood HR estimate was obtained because all deaths occurred in the low-expression group. Subtype-specific analyses were considered exploratory and did not constitute a formal test of differences in prognostic associations between molecular subtypes.

Associations between CpG methylation beta values and *MATR3* mRNA expression were assessed using Pearson’s correlation coefficient. Gene co-expression analyses used Spearman’s rank correlation coefficient. The datasets, gene-selection procedures, functional enrichment methods, and STRING network analyses are described in Section 4.4.

Multiplicity was addressed using the Benjamini–Hochberg procedure within defined families of comparisons. Clinicopathological associations were adjusted separately across nine comparisons in the institutional cohort and three comparisons in the TCGA cohort, with each characteristic contributing one overall *p*-value. Nominal *p*-values are presented in Table 1 and Table 2, and adjusted values in Supplementary Table S1. The two overall-cohort log-rank comparisons were adjusted together. A separate adjustment covered the three estimable subtype-specific log-rank comparisons involving MSI-H, CN-low, and CN-high tumours; the POLE subgroup was excluded from this family because no log-rank *p*-value could be estimated. Subtype-specific patient numbers, event counts, and nominal and adjusted *p*-values are provided in Supplementary Table S2. Multiplicity procedures for the bioinformatic analyses are described in Section 4.4.

All tests for group differences and correlations were two-sided. For analyses incorporating multiplicity adjustment, statistical significance was defined as an adjusted *p*-value < 0.05. Otherwise, *p*-values were reported as nominal, with *p* < 0.05 used as the nominal significance threshold. Cox regression *p*-values and CIs were not adjusted for multiple model evaluations or for the original outcome-driven threshold selection. Multiplicity adjustments for the log-rank comparisons likewise did not account for threshold optimisation.

## 5. Conclusions

Low MATR3 protein expression was associated with shorter overall survival in the institutional EC cohort, including after adjustment for the clinicopathological factors included in the model. The TCGA transcriptomic analysis provided complementary evidence of an association between *MATR3* mRNA expression and survival. Together, these findings support MATR3 as a candidate prognostic biomarker in EC, requiring independent validation before clinical application. Co-expression and enrichment analyses identified nuclear RNA-processing and transcriptional annotations among positively correlated genes and mitochondrial annotations among negatively correlated genes. These patterns generate hypotheses requiring experimental validation and do not establish a molecular mechanism linking MATR3 to these processes. Whether MATR3-related biology could inform future therapeutic strategies remains an open research question requiring functional and preclinical investigation.

## 6. Simple Summary

Matrin-3 (MATR3) is a nuclear matrix protein whose role in endometrial carcinoma (EC) has never been investigated. We studied MATR3 protein expression using immunohistochemistry in 112 patients with endometrial carcinoma and analysed transcriptomic data from an independent exploratory TCGA transcriptomic cohort comprising 172 endometrial carcinoma cases. We found that MATR3 protein expression was lower in endometrial carcinoma than in normal endometrium and that low protein expression was associated with shorter overall survival. *MATR3* mRNA expression was also reduced in tumour tissues, whereas higher mRNA expression was associated with improved survival. Genes correlated with *MATR3* mRNA expression were associated with processes involving nuclear RNA regulation and mitochondrial function. These patterns do not show that MATR3 controls these processes, and experimental studies are needed to investigate their biological significance. This is the first study investigating MATR3 in endometrial carcinoma and provides a foundation for further studies evaluating its prognostic and biological significance.

## Figures and Tables

**Table 1:** Relationship between MATR3 expression and clinicopathological features in EC patients.

Variables	Number (%) *n* = 112	MATR3 Low *n* = 62 (%)	MATR3 High *n* = 50 (%)	Nominal *p*-Value
Age				
≤60	37 (33.04)	21 (56.76)	16 (43.24)	>0.9999
>60	75 (66.96)	41 (54.67)	34 (45.33)	
Histological grade				
G1	7 (6.25)	3 (42.86)	4 (57.14)	0.4973
G2	63 (56.25)	33 (52.38)	30 (47.62)	
G3	42 (37.50)	26 (61.90)	16 (38.10)	
pT status				
T1–T2	93 (83.04)	47 (50.54)	46 (49.46)	0.0252
T3–T4	19 (16.96)	15 (78.95)	4 (21.05)	
pN status				
N0	92 (82.14)	46 (50.00)	46 (50.00)	0.0238
N1	20 (17.86)	16 (80.00)	4 (20.00)	
pM status				
M0	101 (90.18)	53 (52.48)	48 (47.52)	0.1076
M1	11 (9.82)	9 (81.82)	2 (18.18)	
Lymphovascular invasion				
Absent	90 (80.36)	49 (54.44)	41 (45.56)	0.8123
Present	22 (19.64)	13 (59.09)	9 (40.91)	
FIGO stage				
I	51 (45.54)	26 (50.98)	25 (49.02)	0.0295
II	28 (25.00)	11 (39.29)	17 (60.71)	
III	23 (20.54)	17 (73.91)	6 (26.09)	
IV	10 (8.93)	8 (80.00)	2 (20.00)	
Histological type				
Type I	89 (79.46)	51 (57.30)	38 (42.70)	0.4837
Type II	23 (20.54)	11 (47.83)	12 (52.17)	
Myometrial invasion				
<50%	34 (30.36)	18 (52.94)	16 (47.06)	0.8368
≥50%	78 (69.64)	44 (56.41)	34 (43.59)	

Reported *p*-values are nominal (unadjusted). Benjamini–Hochberg-adjusted *p*-values for the nine comparisons are provided in Supplementary Table S1A; none met the adjusted significance threshold of 0.05.

**Table 2:** Relationship between *MATR3* mRNA expression and clinicopathological features in the TCGA cohort.

Variables	Number (%) *n* = 172	*MATR3* High *n* = 97 (%)	*MATR3* Low *n* = 75 (%)	Nominal *p*-Value
Age				
≤60	53 (30.81)	32 (60.38)	21 (39.62)	0.4228
>60	119 (69.19)	65 (54.62)	54 (45.38)	
Histological grade				
G1	13 (7.56)	7 (53.85)	6 (46.15)	0.8991
G2	21 (12.21)	11 (52.38)	10 (47.62)	
G3	138 (80.23)	79 (57.25)	59 (42.75)	
Histological type				
Type I	102 (59.3)	59 (57.84)	43 (42.16)	0.6439
Type II	70 (40.7)	38 (54.29)	32 (45.71)	

Reported *p*-values are nominal (unadjusted). Benjamini–Hochberg-adjusted *p*-values for the three comparisons are provided in Supplementary Table S1B; none met the adjusted significance threshold of 0.05.

**Table 3:** Univariable and multivariable Cox regression analyses of overall survival in our cohort.

Variable	Univariable HR	95% CI	*p*-Value	Multivariable Analysis ^a^: Adjusted HR	95% CI	*p*-Value
MATR3 low vs. high	2.223	1.284–3.848	**0.0043**	2.296	1.284–4.104	**0.0050**
Age > 60 vs. ≤60	3.275	1.658–6.468	**0.0006**	3.272	1.614–6.633	**0.0010**
Histological grade G3 vs. G1–G2	2.166	1.304–3.598	**0.0028**	1.582	0.884–2.829	0.1222
pT T3–T4 vs. T1–T2	1.567	0.848–2.899	0.1519	0.733	0.354–1.520	0.4041
pN N1 vs. N0	1.363	0.723–2.568	0.3378	0.976	0.444–2.145	0.9523
pM M1 vs. M0	1.831	0.826–4.057	0.1363	1.262	0.463–3.445	0.6492
LVSI present vs. absent	2.765	1.569–4.871	**0.0004**	—	—	—

All analyses included 112 patients and 60 deaths. Low MATR3 expression was defined as an H-score < 158 and high expression as an H-score ≥ 158. ^a^ The multivariable Cox model simultaneously included MATR3 expression, age, histological grade, pT, pN, and pM and was stratified by LVSI status. Separate baseline hazards were therefore allowed for patients with and without LVSI, and no adjusted HR was estimated for LVSI (—). Tied event times were handled using Efron’s method. Reported *p*-values are nominal, two-sided Wald *p*-values; values < 0.05 are shown in bold. HR, hazard ratio; CI, confidence interval; LVSI, lymphovascular space invasion.

**Table 4:** Top 50 genes showing the strongest positive correlation with *MATR3* (Spearman rank-order analysis).

*MATR3* (+) Correlated Gene	Cytoband	Spearman’s Correlation	Nominal *p*-Value	*MATR3* (+) Correlated Gene	Cytoband	Spearman’s Correlation	Nominal *p*-Value
*BCLAF1*	6q23.3	0.785	2.98 × 10^−111^	*CHML*	1q43	0.687	8.23 × 10^−75^
*TOP2B*	3p24.2	0.745	2.31 × 10^−94^	*SCAF8*	6q25.2	0.686	1.13 × 10^−74^
*MBTD1*	17q21.33	0.732	1.04 × 10^−89^	*DDX46*	5q31.1	0.685	2.92 × 10^−74^
*ZBTB33*	Xq24	0.721	8.72 × 10^−86^	*APC*	5q22.2	0.684	4.67 × 10^−74^
*ZNF644*	1p22.2	0.720	3.49 × 10^−85^	*LANCL1*	2q34	0.684	5.28 × 10^−74^
*APPBP2*	17q23.2	0.719	6.02 × 10^−85^	*GPALPP1*	13q14.12	0.684	5.48 × 10^−74^
*DDX6*	11q23.3	0.718	1.24 × 10^−84^	*CEP57*	11q21	0.684	6.15 × 10^−74^
*SMAD5*	5q31.1	0.718	1.59 × 10^−84^	*DCAF17*	2q31.1	0.684	8.45 × 10^−74^
*UBTD2*	5q35.1	0.714	2.73 × 10^−83^	*DCP2*	5q22.2	0.683	1.09 × 10^−73^
*RBM12*	20q11.22	0.712	8.49 × 10^−83^	*TRUB1*	10q25.3	0.682	2.07 × 10^−73^
*UBLCP1*	5q33.3	0.712	1.32 × 10^−82^	*SIRT1*	10q21.3	0.682	2.51 × 10^−73^
*CTDSPL2*	15q15.3-q21.1	0.711	3.39 × 10^−82^	*ZNF146*	19q13.1	0.680	7.21 × 10^−73^
*CSNK1G3*	5q23.2	0.711	3.64 × 10^−82^	*MFSD14A*	1p21.2	0.680	9.47 × 10^−73^
*GABPA*	21q21.3	0.707	7.17 × 10^−81^	*PHF6*	Xq26.2	0.677	6.49 × 10^−72^
*CREB1*	2q33.3	0.705	2.40 × 10^−80^	*C11ORF58*	11p15.2	0.675	2.25 × 10^−71^
*PPIP5K2*	5q21.1	0.703	1.27 × 10^−79^	*AGGF1*	5q13.3	0.674	3.63 × 10^−71^
*RBM27*	5q32	0.701	4.25 × 10^−79^	*NUDT21*	16q13	0.673	6.70 × 10^−71^
*G3BP1*	5q33.1	0.700	6.70 × 10^−79^	*TNPO1*	5q13.2	0.672	1.24 × 10^−70^
*GMFB*	14q22.2	0.700	7.80 × 10^−79^	*STAG2*	Xq25	0.672	1.87 × 10^−70^
*CNOT8*	5q33.2	0.700	9.32 × 10^−79^	*MED13*	17q23.2	0.672	2.19 × 10^−70^
*PRPF40A*	2q23.3	0.699	1.49 × 10^−78^	*SRSF10*	1p36.11	0.671	2.36 × 10^−70^
*SP3*	2q31.1	0.694	6.43 × 10^−77^	*SMARCAD1*	4q22.3	0.671	3.03 × 10^−70^
*RASA1*	5q14.3	0.694	8.28 × 10^−77^	*WDR36*	5q22.1	0.671	3.39 × 10^−70^
*C5ORF24*	5q31.1	0.693	1.50 × 10^−76^	*CUL5*	11q22.3	0.668	2.87 × 10^−69^
*SPAST*	2p22.3	0.689	1.61 × 10^−75^	*ZFP91*	11q12.1	0.667	3.21 × 10^−69^

**Table 5:** Top 50 genes showing the strongest negative correlation with *MATR3* (Spearman rank-order analysis).

*MATR3* (−) Correlated Gene	Cytoband	Spearman’s Correlation	Nominal *p*-Value	*MATR3* (−) Correlated Gene	Cytoband	Spearman’s Correlation	Nominal *p*-Value
*TRAPPC5*	19p13.2	−0.690	1.04 × 10^−75^	*DGKZ*	11p11.2	−0.613	1.21 × 10^−55^
*FASTK*	7q36.1	−0.687	9.41 × 10^−75^	*ZDHHC24*	11q13.2	−0.612	1.74 × 10^−55^
*MRPL53*	2p13.1	−0.655	6.89 × 10^−66^	*TREX1*	3p21.31	−0.609	7.93 × 10^−55^
*RNPEPL1*	2q37.3	−0.651	8.36 × 10^−65^	*TAF10*	11p15.4	−0.609	1.01 × 10^−54^
*SSNA1*	9q34.3	−0.650	1.02 × 10^−64^	*NDUFA3*	19q13.42	−0.607	2.80 × 10^−54^
*ANTKMT*	16p13.3	−0.650	1.21 × 10^−64^	*PSMG4*	6p25.2	−0.606	3.43 × 10^−54^
*TMEM11*	17p11.2	−0.640	4.12 × 10^−62^	*ADRM1*	20q13.33	−0.606	3.48 × 10^−54^
*PEX16*	11p11.2	−0.640	4.80 × 10^−62^	*SDF2L1*	22q11.21	−0.606	3.86 × 10^−54^
*GPX4*	19p13.3	−0.638	1.29 × 10^−61^	*MRPL23*	11p15.5	−0.603	1.66 × 10^−53^
*E4F1*	16p13.3	−0.636	5.35 × 10^−61^	*MRPL55*	1q42.13	−0.603	1.74 × 10^−53^
*NDUFS7*	19p13.3	−0.636	5.71 × 10^−61^	*LIN37*	19q13.12	−0.602	2.56 × 10^−53^
*MIF*	22q11.23	−0.633	2.38 × 10^−60^	*POLR2I*	19q13.12	−0.602	2.90 × 10^−53^
*SMIM29*	6p21.31	−0.632	4.14 × 10^−60^	*CCM2*	7p13	−0.600	6.67 × 10^−53^
*ROMO1*	20q11.22	−0.632	4.59 × 10^−60^	*FBXL15*	10q24.32	−0.600	7.23 × 10^−53^
*ELOB*	16p13.3	−0.630	1.21 × 10^−59^	*UQCR10*	22q12.2	−0.600	9.31 × 10^−53^
*SLC66A1*	1p36.13	−0.630	1.39 × 10^−59^	*FAM50A*	Xq28	−0.600	9.52 × 10^−53^
*NDUFS8*	11q13.2	−0.629	1.77 × 10^−59^	*SIPA1*	11q13.1	−0.599	1.13 × 10^−52^
*NDUFAF3*	3p21.31	−0.622	9.35 × 10^−58^	*ANKRD13D*	11q13.2	−0.599	1.16 × 10^−52^
*JMJD8*	16p13.3	−0.621	1.29 × 10^−57^	*ABHD17A*	19p13.3	−0.599	1.32 × 10^−52^
*CPTP*	1p36.33	−0.620	3.05 × 10^−57^	*CIAO2B*	16q22.1	−0.598	2.30 × 10^−52^
*SHARPIN*	8q24.3	−0.619	5.89 × 10^−57^	*EXOSC4*	8q24.3	−0.597	3.65 × 10^−52^
*TRAPPC1*	17p13.1	−0.618	6.95 × 10^−57^	*SELENOO*	22q13.33	−0.596	4.68 × 10^−52^
*BLOC1S1*	12q13.2	−0.617	1.54 × 10^−56^	*ACOT8*	20q13.12	−0.596	6.73 × 10^−52^
*MRPL41*	9q34.3	−0.616	1.79 × 10^−56^	*VPS28*	8q24.3	−0.595	6.96 × 10^−52^
*GUK1*	1q42.13	−0.615	4.92 × 10^−56^	*IDH3G*	Xq28	−0.595	7.15 × 10^−52^

## Data Availability

The datasets generated during and/or analyzed during the current study are available from the corresponding author on reasonable request.

## Supplementary Materials

The following supporting information can be downloaded at: https://www.mdpi.com/article/10.3390/ijms27219708/s1.

## Abbreviations

The following abbreviations are used in this manuscript:

**Abbreviation**	**Definition**
EC	Endometrial carcinoma
MATR3	Matrin-3
TCGA	The Cancer Genome Atlas
OS	Overall survival
HR	Hazard ratio
CI	Confidence interval
IHC	Immunohistochemistry
FFPE	Formalin-fixed paraffin-embedded
RBP	RNA-binding protein
PPI	Protein–protein interaction
GO	Gene Ontology
BLBC	Basal-like breast cancer
HCC	Hepatocellular carcinoma
ccRCC	Clear cell renal cell carcinoma
NSCLC	Non-small cell lung cancer
DSB	Double-strand break
EMT	Epithelial-to-mesenchymal transition
ALS	Amyotrophic lateral sclerosis
FTD	Frontotemporal dementia
AJCC	American Joint Committee on Cancer
