# Supplementary material for: Low MATR3 Expression Is Associated with Poor Prognosis in Endometrial Carcinoma: An Integrated Immunohistochemical and Transcriptomic Analysis
Source: Int J Mol Sci. 2026 Oct 29;27(21):9708. doi: 10.3390/ijms27219708 (PMC13763437; doi:10.3390/ijms27219708)
Supplement: Supplementary file 1 [file ijms-27-09708-s001.zip › ijms-4603549-supplementary.pdf]

Supplementary Materials

Low MATR3 Expression Is Associated with Poor Prognosis in Endometrial Carcinoma: An Integrated Immunohistochemical and Transcriptomic Analysis

Jonasz Podemski, Dariusz Grzanka, Edyta Podemska, Natalia Skoczylas-Makowska, Dominika Jerka, Maciej Gagat and Paulina Antosik

Supplementary Table S1. Nominal and Benjamini–Hochberg-adjusted p-values for associations between MATR3 expression and clinicopathological characteristics.

Clinicopathological characteristic	Nominal p-value	BH-adjusted p-value
**A. Institutional IHC cohort (n = 112; nine comparisons)**		
Age	>0.9999	1.0000*
Histological grade	0.4973	0.7460
pT status	0.0252	0.0885
pN status	0.0238	0.0885
pM status	0.1076	0.2421
Lymphovascular space invasion	0.8123	0.9414
FIGO stage	0.0295	0.0885
Histological type	0.4837	0.7460
Myometrial invasion	0.8368	0.9414
**B. TCGA transcriptomic cohort (n = 172; three comparisons)**		
Age	0.4228	0.8991
Histological grade	0.8991	0.8991
Histological type	0.6439	0.8991

Notes. Associations concern low versus high MATR3 protein expression in the institutional cohort and low versus high MATR3 mRNA expression in the TCGA cohort. Benjamini–Hochberg (BH) adjustment was performed separately across the nine comparisons in panel A and the three comparisons in panel B. Each characteristic contributed one overall p-value; repeated p-values across category rows in Tables 1 and 2 were not counted as separate tests. No association met the BH-adjusted significance threshold of 0.05.

Adjustment was performed during manuscript revision using the nominal p-values reported in Tables 1 and 2, rounded to four decimal places. Adjusted p-values are therefore approximate and are presented to four decimal places.

* For age in panel A, the reported nominal p-value >0.9999 was entered as 1.0000 for calculation; this does not affect the adjusted p-values for the other comparisons.

Abbreviations. BH, Benjamini–Hochberg; IHC, immunohistochemistry; TCGA, The Cancer Genome Atlas; FIGO, International Federation of Gynecology and Obstetrics.

Supplementary Table S2. Overall survival comparisons between low and high MATR3 mRNA expression groups within TCGA-UCEC molecular subtypes.

Molecular subtype	Total n	Deaths	Low expression n (deaths)	High expression n (deaths)	Nominal p-value	BH-adjusted p-value	Univariable HR (95% CI)

POLE	15	0	3 (0)	12 (0)	NE	NE	NE
MSI-H	39	5	21 (5)	18 (0)	0.0656	0.0983	NE
CN-low	30	2	11 (2)	19 (0)	0.0461	0.0983	NE
CN-high	82	22	38 (11)	44 (11)	0.6791	0.6791	1.19 (0.51–2.77)

Notes. Molecular subtype annotation was available for 166 of the 172 TCGA-UCEC cases. Six cases without subtype annotation were excluded only from subtype-specific analyses. Overall survival was measured in months, with death as the event and surviving patients censored at their recorded follow-up time. Hazard ratios compare low with high MATR3 expression and were estimated using univariable Cox proportional hazards models with Efron handling of tied event times and 95% Wald confidence intervals. For HR estimates, NE indicates that estimation was not possible because no deaths occurred in the POLE subgroup, or that no finite maximum partial-likelihood estimate was obtained because all deaths occurred in the low-expression group in the MSI-H and CN-low subgroups. The reported p-values refer to the log-rank comparisons, not to Cox Wald tests.

Low expression was defined as MATR3 <10.17 and high expression as MATR3 ≥10.17. The same previously selected cut-off was applied to all subtypes without subtype-specific re-estimation. Counts in parentheses indicate deaths.

Two-sided, unweighted log-rank tests were recalculated in R using survival::survdiff (rho = 0), with p-values obtained from the asymptotic chi-square distribution with one degree of freedom. Benjamini–Hochberg (BH) adjustment was applied to the full-precision p-values of the three estimable subtype comparisons. Values are displayed to four decimal places. No comparison met the BH-adjusted significance threshold of 0.05.

NE, not estimable: no deaths occurred in the POLE subgroup. No nominal p-value was assigned to this subgroup, and it was not included in the BH adjustment.

The analyses are exploratory, particularly given the small number of events in some subgroups. BH adjustment accounts for the three subtype comparisons but does not account for the original survival-based selection of the expression cut-off. Subgroup-specific p-values do not constitute a formal test of differences in the prognostic association between subtypes.

Abbreviations: TCGA, The Cancer Genome Atlas; UCEC, uterine corpus endometrial carcinoma; CN, copy number; MSI-H, microsatellite instability-high; POLE, polymerase epsilon; BH, Benjamini–Hochberg.

Supplementary Table S3. Comparison of *MATR3* mRNA expression across TCGA molecular subtypes of endometrial carcinoma.

Subtype	n	median	IQR	mean	SD	min	max
POLE	15	10.49	10.350–10.825	10.405	0.586	9.102	11.15
MSI-H	39	10.12	9.962–10.440	10.178	0.437	9.227	11.4
CN-low	30	10.41	10.012–10.660	10.407	0.622	8.916	12.09
CN-high	82	10.24	9.829–10.565	10.266	0.73	8.394	12.74

Supplementary Table S4. Exploratory comparison of LVSI-stratified clinical Cox models with and without MATR3 protein expression.

Measure	Clinical model	Clinical model + MATR3
Patients / deaths	112 / 60	112 / 60
Regression coefficients	5	6
Log partial likelihood	-212.015	-207.893
Partial-likelihood AIC	434.029	427.786
Apparent Harrell's C within LVSI strata	0.6917	0.7232

The clinical model included age (>60 vs. ≤60 years), histological grade (G3 vs. G1–G2), pT (T3–T4 vs. T1–T2), pN (N1 vs. N0), and pM (M1 vs. M0). The extended model additionally included MATR3 expression (low, H-score <158, vs. high, H-score ≥158). Both models were stratified by LVSI status, fitted to the same 112 complete cases with 60 deaths, and used Efron's method for tied event times. No covariate selection was performed for this comparison.

The nested-model partial likelihood-ratio test yielded $\chi^2 = 8.243$ with 1 degree of freedom (nominal $p = 0.0041$). The change in AIC (extended minus clinical) was −6.243. The change in apparent C was +0.0315. Lower AIC indicates better relative fit after penalizing model complexity.

Harrell's C was calculated using comparable patient pairs within each LVSI stratum, with counts pooled across strata; between-stratum pairs were not evaluated. Values describe apparent discrimination in the development cohort and were not corrected for optimism. No inferential test of the change in C was performed. The original outcome-driven MATR3 cut-off was held fixed; neither the likelihood-ratio p-value nor the discrimination estimates account for its selection. These exploratory comparisons do not establish external predictive performance or clinical utility.

AIC, Akaike information criterion; LVSI, lymphovascular space invasion. Software: R 4.4.3, survival 3.8-6.

Supplementary Table S5

Selected Reactome and Gene Ontology enrichment results for genes correlated with MATR3. Analyses were repeated on 19 October 2026. All terms with BH-adjusted p <0.05 are included, together with selected previously highlighted terms that did not meet this threshold. Complete unfiltered outputs are supplied in Supplementary Data S1.

A Reactome pathways

Gene set	Pathway	Nominal p	BH-adjusted p
Positive	mRNA Polyadenylation R-HSA-9770562	1.32e-04	0.0199
Positive	Regulation of MITF-M dependent genes involved in metabolism R-HSA-9854907	1.49e-04	0.0199
Positive	Metabolism of RNA R-HSA-8953854	1.80e-04	0.0199
Positive	mRNA 3'-end processing R-HSA-72187	2.83e-04	0.0235
Positive	MECP2 regulates transcription factors R-HSA-9022707	5.91e-04	0.0390
Positive	Deadenylation-dependent mRNA decay R-HSA-429914	0.0017	0.0955
Negative	Complex I biogenesis R-HSA-6799198	9.68e-05	0.0381
Negative	Mitochondrial translation elongation R-HSA-5389840	4.57e-04	0.0452
Negative	Mitochondrial translation initiation R-HSA-5368286	4.90e-04	0.0452
Negative	Respiratory electron transport R-HSA-611105	5.05e-04	0.0452
Negative	Mitochondrial ribosome-associated quality control R-HSA-9937383	5.79e-04	0.0452
Negative	Mitochondrial translation termination R-HSA-5419276	7.22e-04	0.0470

Reactome release 97; human projection; no additional interactors; disease pathways retained; default reference background. Adjusted p-values were obtained from Reactome (entities FDR). Mapping: 30/50 submitted identifiers in the positive set and 40/50 in the negative set; 294 and 386 pathways were returned, respectively. Significance was assessed at adjusted $p < 0.05$.

B Gene Ontology positively correlated genes

Category	GO term	Genes	Nominal p	BH-adjusted p
BP	GO:0000281<br>mitotic cytokinesis	3	0.0113	0.7255
CC	GO:0005634<br>nucleus	39	1.09e-10	8.05e-09
CC	GO:0005654<br>nucleoplasm	30	1.29e-10	8.05e-09
CC	GO:0005730<br>nucleolus	10	2.44e-04	0.0102
CC	GO:0000792<br>heterochromatin	4	3.48e-04	0.0109
MF	GO:0003723<br>RNA binding	16	2.83e-06	4.73e-04
MF	GO:0003676<br>nucleic acid binding	9	1.48e-04	0.0124
MF	GO:0003729<br>mRNA binding	6	7.02e-04	0.0391

DAVID Knowledgebase v2026_2; direct GO annotations; default Homo sapiens background. Nominal p-values are EASE scores (modified Fisher exact test). BH adjustment was performed separately within each ontology category and gene set using all returned terms, not only the selected rows shown here. Count threshold 1 and EASE threshold 1 were used for complete reporting. BP, biological process; CC, cellular component; MF, molecular function. Genes denotes the number of list genes annotated to the term.

Fifty submitted genes were recognised. There were 384 BP, 125 CC and 167 MF terms. No BP term was significant after adjustment; four CC and three MF terms met adjusted p <0.05. Category-specific annotated list totals were BP 47, CC 49 and MF 49.

C Gene Ontology negatively correlated genes

Category	GO term	Genes	Nominal p	BH-adjusted p
BP	GO:0009060<br>aerobic respiration	4	6.08e-04	0.1721
CC	GO:0005739<br>mitochondrion	18	1.02e-07	1.15e-05
CC	GO:0005743<br>mitochondrial inner membrane	11	3.72e-07	2.10e-05
CC	GO:0005762<br>mitochondrial large ribosomal subunit	4	2.52e-04	0.0095
MF	GO:0005515<br>protein binding	45	8.12e-04	0.0983

DAVID Knowledgebase v2026_2; direct GO annotations; default Homo sapiens background. Nominal p-values are EASE scores (modified Fisher exact test). BH adjustment was performed separately within each ontology category and gene set using all returned terms, not only the selected rows shown here. Count threshold 1 and EASE threshold 1 were used for complete reporting. BP, biological process; CC, cellular component; MF, molecular function. Genes denotes the number of list genes annotated to the term.

Fifty submitted genes were recognised. There were 283 BP, 113 CC and 121 MF terms. No BP or MF term was significant after adjustment; three CC terms met adjusted $p < 0.05$. Aerobic respiration and protein binding are included to document their nominal-only significance.

Supplementary Table S6. Clinicopathological characteristics of the institutional endometrial carcinoma cohort according to MATR3 protein expression.

Variables	Number (%) n=112	MATR3 low n=62 (%)	MATR3 high n=50 (%)
**Age**			
≤60	37 (33.04)	21 (56.76)	16 (43.24)
>60	75 (66.96)	41 (54.67)	34 (45.33)
**Histological grade**			
G1	7 (6.25)	3 (42.86)	4 (57.14)
G2	63 (56.25)	33 (52.38)	30 (47.62)
G3	42 (37.50)	26 (61.90)	16 (38.10)
**pT status**			
T1-T2	93 (83.04)	47 (50.54)	46 (49.46)
T3-T4	19 (16.96)	15 (78.95)	4 (21.05)
**pN status**			
N0	92 (82.14)	46 (50.00)	46 (50.00)
N1	20 (17.86)	16 (80.00)	4 (20.00)
**pM status**			
M0	101 (90.18)	53 (52.48)	48 (47.52)
M1	11 (9.82)	9 (81.82)	2 (18.18)
**Lymphovascular invasion**			
Absent	90 (80.36)	49 (54.44)	41 (45.56)
Present	22 (19.64)	13 (59.09)	9 (40.91)
**FIGO stage**			
I	51 (45.54)	26 (50.98)	25 (49.02)
II	28 (25.00)	11 (39.29)	17 (60.71)
III	23 (20.54)	17 (73.91)	6 (26.09)
IV	10 (8.93)	8 (80.00)	2 (20.00)
**Histological type**			
Type I	89 (79.46)	51 (57.30)	38 (42.70)
Type II	23 (20.54)	11 (47.83)	12 (52.17)
**Myometrial invasion**			
<50%	34 (30.36)	18 (52.94)	16 (47.06)
≥50%	78 (69.64)	44 (56.41)	34 (43.59)

Data are presented as n (%). Percentages in the overall "Number (%)" column are calculated using all 112 patients as the denominator. Percentages in the low- and high-expression columns are calculated within each clinicopathological category, using the category total reported in the same row as the denominator, rather than the expression-group totals of 62 and 50. All listed clinicopathological variables were available for all 112 patients; there were zero missing observations for each variable.

Supplementary Table S7. Clinicopathological characteristics of the TCGA endometrial carcinoma cohort according to *MATR3* mRNA expression.

Variables	Number (%) n=172	MATR3 high n=97 (%)	MATR3 low n=75 (%)
**Age**			
≤60	53 (30.81)	32 (60.38)	21 (39.62)
>60	119 (69.19)	65 (54.62)	54 (45.38)
**Histological grade**			
G1	13 (7.56)	7 (53.85)	6 (46.15)
G2	21 (12.21)	11 (52.38)	10 (47.62)
G3	138 (80.23)	79 (57.25)	59 (42.75)
**Histological type**			
Type I	102 (59.3)	59 (57.84)	43 (42.16)
Type II	70 (40.7)	38 (54.29)	32 (45.71)

Data are presented as n (%). Percentages in the overall "Number (%)" column are calculated using all 172 patients as the denominator. Percentages in the high- and low-expression columns are calculated within each clinicopathological category, using the category total reported in the same row as the denominator, rather than the expression-group totals of 97 and 75. Age, histological grade, and histological type were available for all 172 patients; there were zero missing observations for each variable.

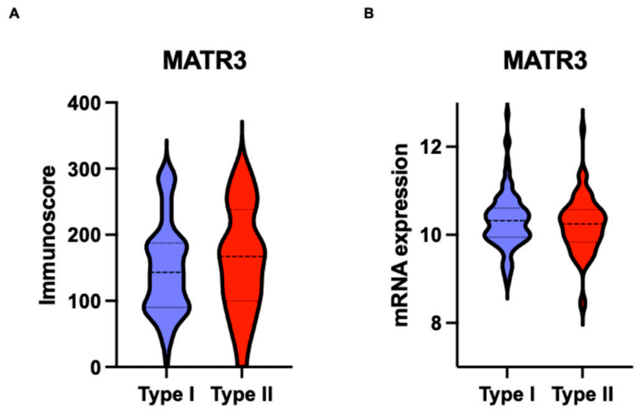

Supplementary Figure S1. Comparison of MATR3 expression between histological subtypes of endometrial carcinoma. **(A)** MATR3 protein expression assessed by H-score in the immunohistochemical cohort: type I carcinomas, n = 89; type II carcinomas, n = 23. **(B)** *MATR3* mRNA expression in the TCGA cohort: type I carcinomas, n = 102; type II carcinomas, n = 70. No statistically significant differences were observed between type I and type II carcinomas in either the protein-expression cohort or the mRNA-expression cohort. P-values were calculated using the two-tailed Mann–Whitney U test. Dashed lines indicate medians, and dotted lines indicate quartiles.

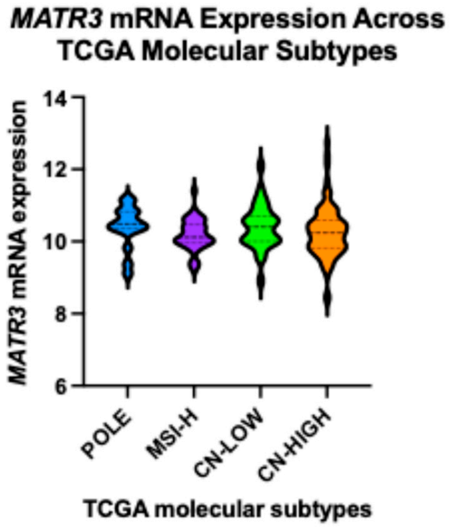

Supplementary Figure S2. *MATR3* mRNA expression across TCGA molecular subtypes of endometrial carcinoma. *MATR3* mRNA expression was compared among POLE, MSI-H, CN-low, and CN-high tumours in the TCGA-UCEC cohort. Molecular subtype annotation was available for 166 cases: POLE, n = 15; MSI-H, n = 39; CN-low, n = 30; and CN-high, n = 82. No statistically significant differences in *MATR3* expression were observed among the four molecular subtypes (Kruskal–Wallis test: H = 4.581, p = 0.2052).

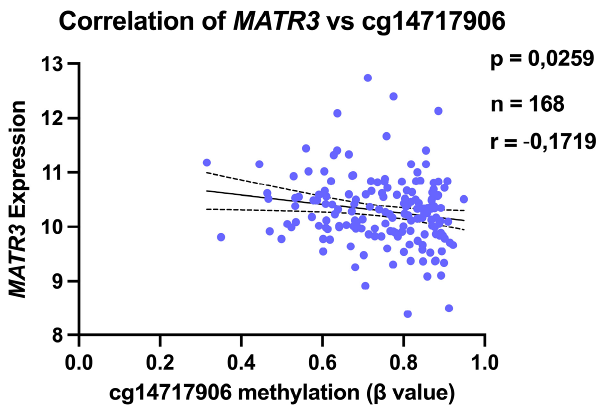

Supplementary Figure S3. Correlation between *MATR3* mRNA expression and cg14717906 methylation in the TCGA-UCEC cohort. DNA methylation beta values for cg14717906 showed a weak negative correlation with *MATR3* mRNA expression (r = −0.172, p = 0.0259; n = 168).

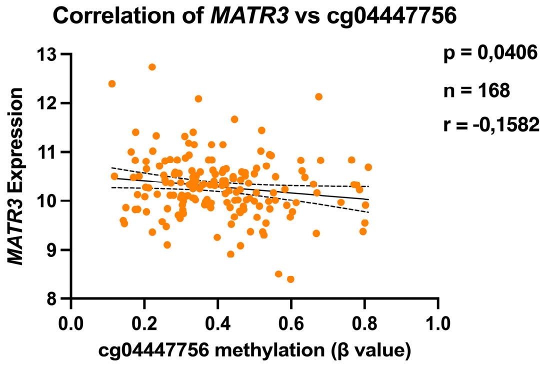

Supplementary Figure S4. Correlation between *MATR3* mRNA expression and cg04447756 methylation in the TCGA-UCEC cohort. DNA methylation beta values for cg04447756 showed a weak negative correlation with *MATR3* mRNA expression (r = −0.158, p = 0.0406; n = 168).

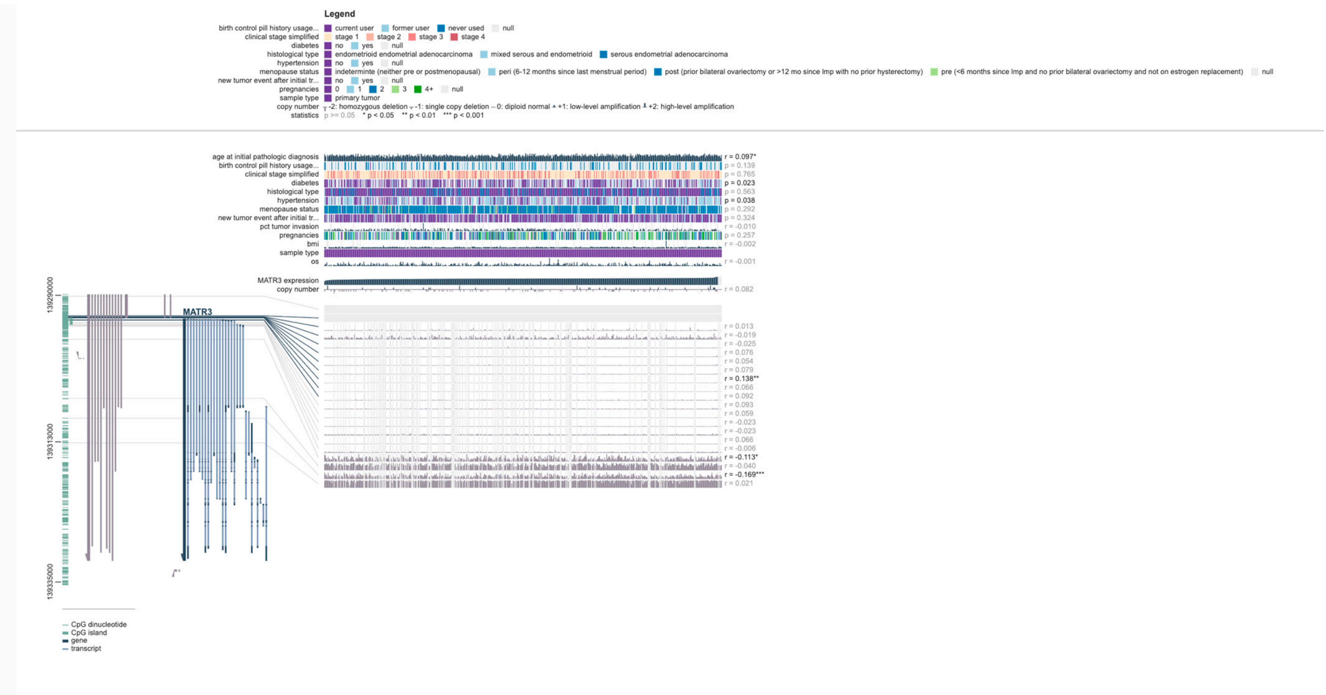

Supplementary Figure S5. Additional MEXPRESS analysis of the MATR3 expression–methylation association in TCGA-UCEC. The MEXPRESS analysis was performed in a larger TCGA-UCEC cohort and included methylation probes across the *MATR3* locus together with clinical and molecular annotations. The association for cg04447756 remained significant after correction for multiple testing, whereas cg14717906 did not show a significant association in this additional analysis.
